# FSTL1 Orchestrates Metabolic‐Epigenetic Crosstalk: Glycolysis‐Dependent H3K18 Lactylation Drives Cartilage Fibrosis in Osteoarthritis

**DOI:** 10.1002/advs.202512002

**Published:** 2025-11-26

**Authors:** Feng Lu, Yunyuan Yu, Guangrong Yin, Huiqun Hu, Shishuo Li, Yuting Tang, Yimin Liu, Maoyuan Li, Liang liang Wang, Chao Xu, Gongyin Zhao, Baojun Zhou, Yuji Wang

**Affiliations:** ^1^ Department of Orthopedics The Third Affiliated Hospital of Nanjing Medical University (Changzhou No. 2 People's Hospital) Changzhou Jiangsu 213003 P. R. China; ^2^ Nanjing Medical University Nanjing 210000 P. R. China; ^3^ Articular Orthopaedics The Third Affiliated Hospital of Soochow University Changzhou 213003 P. R. China; ^4^ Department of Thoracic Radiation Oncology Zhejiang Cancer Hospital Hangzhou 310022 P. R. China; ^5^ Department of Infectious Diseases the Second Affiliated Hospital Zhejiang University School of Medicine Hangzhou 310009 P. R. China; ^6^ Graduate School of Dalian Medical University Dalian Liaoning 116044 P. R. China; ^7^ Department of Medical Genetics Nanjing Medical University Longmian Road 101 Nanjing 211166 P. R. China; ^8^ Jiangsu Key Laboratory of Xenotransplantation Nanjing Medical University Longmian Road 101 Nanjing 211166 P. R. China; ^9^ Department of Orthopedics The Third Affiliated Hospital of Gansu University of Chinese Medicine 222 Silong Road Baiyin 730900 P. R. China; ^10^ Center for bone disease rehabilitation The Third Affiliated Hospital of Nanjing Medical University (Changzhou No. 2 People's Hospital) Changzhou Jiangsu 213003 P. R. China; ^11^ Department of Orthopedic Surgery and Biochemistry and Molecular Biology Mayo Clinic Rochester MN 55905 USA

**Keywords:** cartilage, fibrosis, FSTL1, glycolysis, lactylation, osteoarthrosis

## Abstract

The progression of osteoarthritis (OA) is fundamentally characterized by the aberrant transformation of chondrocytes into a fibrotic phenotype, although the precise molecular mechanisms involved remain inadequately understood. In this study, the interplay between epigenetic modifications and metabolic reprogramming during the activation of fibrocartilage cells in osteoarthritis was investigated. The findings demonstrate that FSTL1 markedly upregulates key glycolytic enzymes, including LDHA, HK2, and PKM, in chondrocytes, triggering a “glycolytic burst” that results in elevated intracellular lactate levels. This accumulated lactate acts as a precursor for epigenetic modifications, specifically promoting the lactylation of histone H3 lysine 18 (H3K18la) in fibrocartilage cells, thereby facilitating the transcriptional activation of critical fibrosis‐related genes such as Itga6, Cxcl10, and Parp16. Notably, pharmacological inhibition of the PI3K/mTOR pathway or lactate dehydrogenase significantly diminishes H3K18la levels and markers of chondrocyte fibrosis, while exogenous lactate supplementation can counteract this effect. In summary, this study unveils the core mechanism by which FSTL1 reshapes the epigenetic landscape of chondrocytes and drives the fibrotic process through the activation of the “glycolysis‐lactate‐H3K18la” cascade axis, offering a dual‐target intervention strategy for OA involving metabolic reprogramming and epigenetic modification.

## Graphical abstract

FSTL1 promotes glycolysis during chondrocyte fibrosis by triggering the HIF‐1 signaling pathway, which causes lactate to accumulate. The buildup of lactate leads to changes in histone lysine lactylation, which in turn enhances the expression of genes associated with fibrosis. Preventing lactate production effectively stops chondrocyte fibrosis, but this effect is reversed by the addition of exogenous lactate.

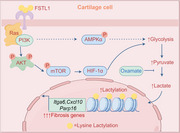

## 1 Introduction

Knee chondrocytes thrive in hypoxic environments by stabilizing glycolysis and producing lactate via hypoxia‐inducible factor 1‐alpha (HIF‐1α), a key regulator of metabolic reprogramming that ensures their survival and function.^[^ ^1^, ^2^, ^3^ ^]^ In osteoarthritis (OA), however, HIF‐1α‐driven metabolic shifts interact with inflammatory pathways, exacerbating cartilage degradation.^[^ ^4^ ^]^ While HIF‐1α enhances glycolysis and lactate accumulation, the broader implications of these metabolic byproducts remain unclear.^[^ ^5^ ^]^

Lactate serves as a multifunctional molecule, functioning not only as an energy substrate and immunomodulatory signal but also as a mediator of histone lactylation—an epigenetic modification involving lactate‐derived lactyl groups that modulate chromatin‐associated gene transcription.^[^ ^6^, ^7^, ^8^ ^]^ Therefore, lactic acid becomes an effective signaling molecule and an epigenetic regulatory factor that directly regulates gene transcription. While lactate is conventionally recognized as a metabolic byproduct of glycolysis, emerging evidence underscores its noncanonical roles in both physiological and pathological contexts, particularly its interplay with histone lactylation in regulating cellular differentiation and fibrotic disease progression.^[^ ^6^, ^9^, ^10^ ^]^ Despite its established metabolic significance, the mechanistic contributions of lactate to epigenetic reprogramming and fibrosis remain incompletely characterized, necessitating further investigation to elucidate its therapeutic potential.^[^ ^7^ ^]^ While lactate has been implicated in the pathogenesis of OA,^[^ ^11^ ^]^ the regulatory function of lactylation in modulating chondrocyte phenotype remains largely unexplored. However, there is a lack of corresponding research on the effects of lactylation modification on chondrocytes, especially in terms of chondrocyte fibrosis.

Hyaline cartilage, despite its biomechanical resilience, exhibits limited regenerative capacity, rendering damage a critical precursor to joint dysfunction, inflammatory amplification, and fibrocartilage deposition—a mechanically inferior tissue that fails to replicate native cartilage function.^[^ ^12^, ^13^, ^14^ ^]^ Consequently, therapeutic strategies must prioritize regenerative repair of hyaline cartilage over compensatory fibrocartilage formation.^[^ ^15^ ^]^ Through the intersection screening of transcriptomics and public databases, we found that Follistatin‐like protein 1 (FSTL1) was significantly upregulated in prefibrotic chondrocytes. FSTL1, an exocrine glycoprotein implicated in inflammatory arthritis, demonstrates paradoxical regulatory effects: it exacerbates chondrocyte apoptosis and inflammatory pathways while concurrently promoting proliferation and fibrosis.^[^ ^16^, ^17^, ^18^, ^19^ ^]^ Preliminary observations from our laboratory suggest FSTL1 induces chondrocyte fibrotic transformation, a phenomenon corroborated by its documented therapeutic relevance in cardiac and hepatic fibrosis.^[^ ^18^, ^20^, ^21^, ^22^ ^]^ In addition, FSTL1 has been shown to enhance glycolysis in macrophages, so we hypothesized that FSTL1 might also enhance the glycolytic capacity of chondrocytes, possibly through the HIF‐1 signaling pathway. However, the molecular mechanisms underlying FSTL1‐mediated chondrocyte fibrosis remain unresolved, highlighting a critical gap in understanding its dualistic role in cartilage homeostasis and disease.

**Scheme 1:**
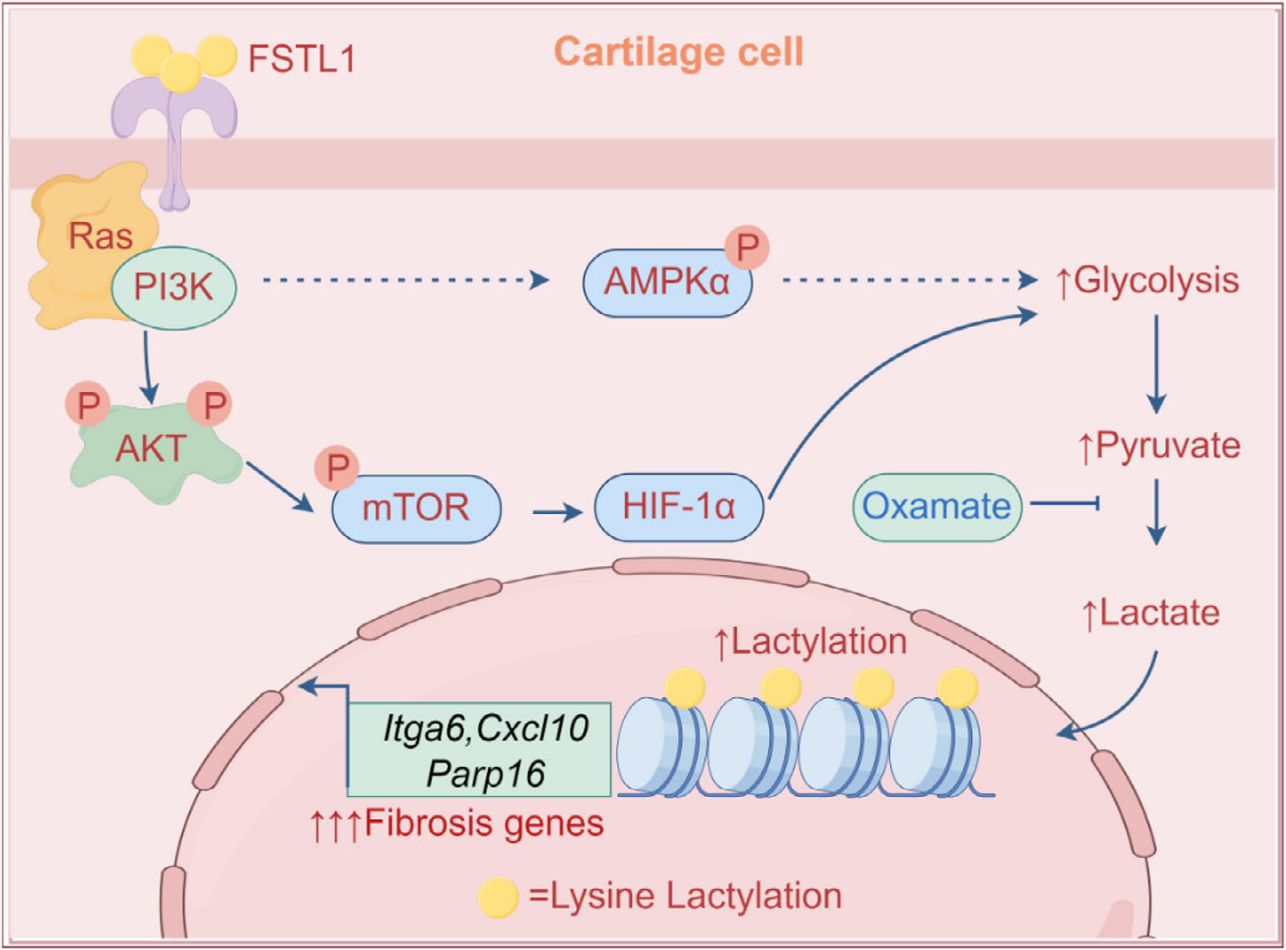
Schematic illustration of FSTL1‐mediated fibrosis induced by chondrocytes. During the process of chondrocyte fibrosis, FSTL1 enhances glycolysis by activating the HIF‐1 signaling pathway, leading to the accumulation of lactic acid. This accumulation of lactic acid induces histone lysine lactylation modification, which in turn promotes the expression of genes related to fibrosis. Inhibiting lactic acid production can effectively prevent chondrocyte fibrosis, while exogenous lactic acid can reverse this effect.

In this study, we demonstrated that FSTL1 induces chondrocyte fibrosis in vitro and in vivo. FSTL1 Improve by promoting HIF‐1α expression in enhances glycolytic activity in chondrocytes, driving lactate accumulation, and facilitating histone lactylation—a lactate‐dependent epigenetic modification. Pharmacological inhibition of lactate synthesis attenuated histone lactylation and suppressed fibrotic transformation, while exogenous lactate supplementation rescued the phenotype. Transcriptomic profiling and CUT&Tag assays further revealed that FSTL1 promotes HIF‐1α‐mediated glycolysis and histone lactylation, thereby establishing a causal link between metabolic reprogramming and chondrocyte fibrosis (Scheme 1). These findings implicate FSTL1 as a therapeutic target for mitigating fibrosis in OA.

## 2 Result

### 2.1 FSTL1 is Involved in the Progression of Osteoarthritis

**Figure 1:**
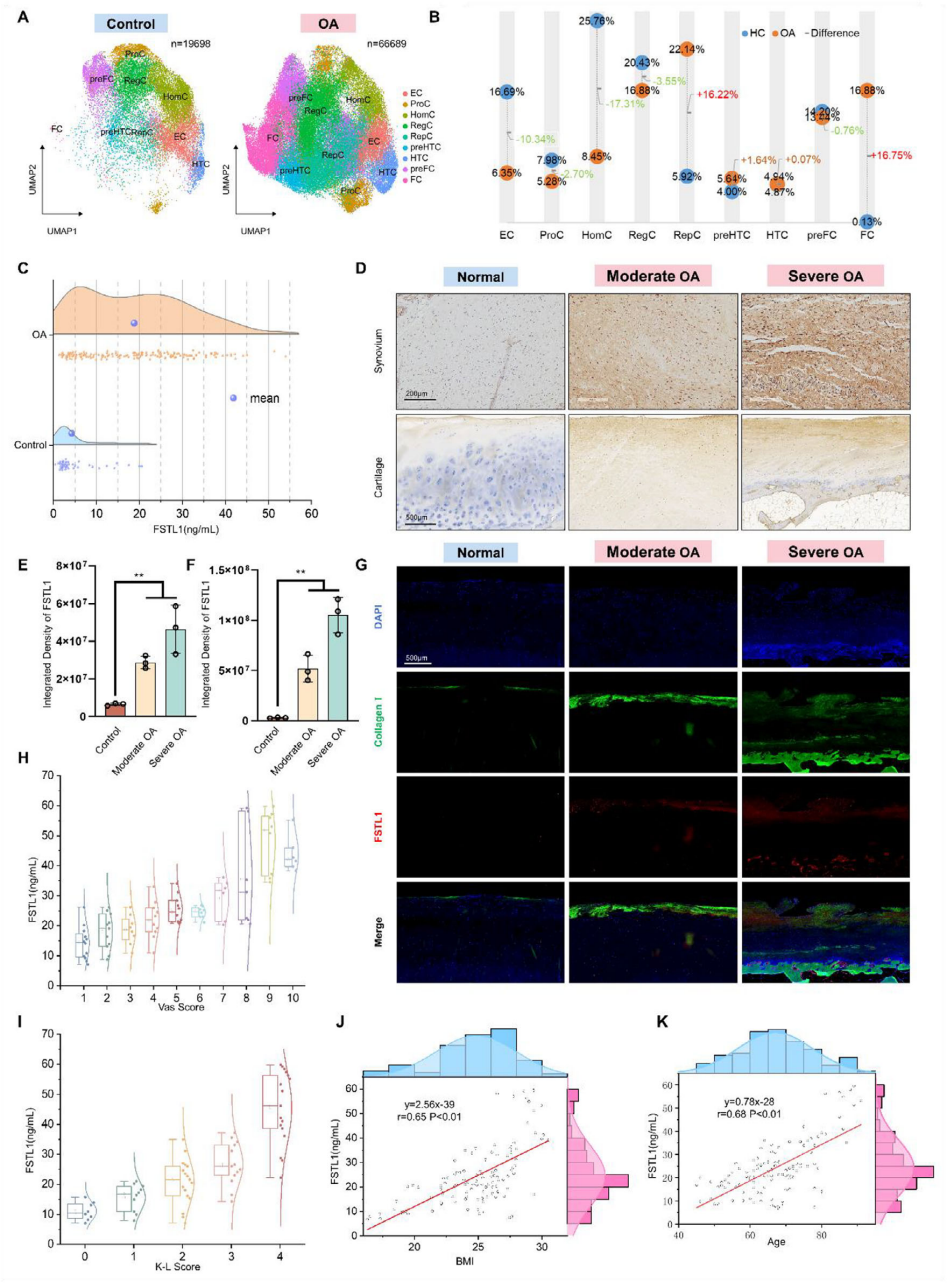
FSTL1 is involved in the progression of osteoarthritis. A,B) Dimensionality reduction of single‐cell sequencing data from chondrocytes of healthy and OA patients, revealing the proportions and variations of different chondrocyte subtypes. Nine distinct chondrocyte populations were identified: EC (effector chondrocytes, red), ProC (proliferation chondrocytes, orange), HomC (homeostasis chondrocytes, brown), RegC (regulator chondrocytes, green), RepC (reparative chondrocytes, aqua), preHTC (prehypertrophic chondrocyte, azure), HTC (hypertrophic chondrocyte, blue), preFC (prefibrocartilage chondrocytes, purple), FC (fibrocartilage chondrocytes, pink). C–G) Comparative analysis of FSTL1 levels in joint fluid, cartilage, and synovial tissue of healthy and OA patients. H–K) Correlation analysis between FSTL1 levels in joint fluid of OA patients and clinical indicators. T‐test, *n* = 3/group, ^*^ *p* < 0.05, ^**^ *p* < 0.01.

To determine whether chondrocyte differentiation states shift during OA pathogenesis, we analyzed single‐cell RNA sequencing (scRNA‐seq) data from knee joint chondrocytes in OA patients versus healthy controls.^[^ ^23^ ^]^ Comparative analysis revealed marked differentiation shifts in OA chondrocyte populations (**Figure** **1**A; Figure S1A,B, Supporting Information), characterized by significant reductions in effector and homeostatic chondrocytes alongside expansions in proliferative and fibro‐chondrocytes (Figure 1B). These findings suggest that fibrosis of chondrocytes is recognized as one of the key pathological mechanisms underlying the progression of OA.

To investigate the relationship between FSTL1 and OA, normal synovial tissues from 30 patients with traumatic injuries during surgery and synovial tissues from 30 OA patients were collected. Additionally, 60 previously collected samples of normal knee joint fluid and 158 samples of OA knee joint fluid were also included in the study.^[^ ^24^ ^]^ According to the results of the ELISA analysis, the OA patients tend to exhibit significantly higher level of FSTL1 in joint fluid in contrast to normal individuals (Figure 1C). To further elucidate the source of FSTL1, immunohistochemical staining was employed to assess the FSTL1 expression in various cartilage and synovial tissues. Compared to normal synovium, the notable elevation of FSTL1 expression was detected in the synovium of OA patients. Correspondingly, the amount of FSTL 1 was also significantly higher in the cartilage of OA patients than in normal cartilage (Figure 1D–F). The cross‐section of cartilage shows that the extracellular matrix near the cartilage surface contains more FSTL1, while the content of FSTL1 gradually decreases as it approaches the base of the cartilage. This phenomenon suggests that FSTL1 in cartilage is more likely to originate from synovial fluid rather than being secreted by chondrocytes themselves.

Immunofluorescence co‐localization experiments demonstrated a high degree of synchronization between collagen I and FSTL1, further supporting this conclusion (Figure 1G). Correlation analysis revealed positive associations between joint fluid FSTL1 levels and patient VAS score, K‐L score, age, and body mass index (BMI) (Figure 1H–K). Higher FSTL1 levels correlated with increased VAS and K‐L scores, reflecting greater OA severity. FSTL1 levels also positively correlated with age and BMI. These data indicate that FSTL1 expression is elevated in OA and associated with disease severity.

### 2.2 FSTL1 Aggravates Cartilage Erosion and Osteophyte Production in DMM‐Induced Mice

**Figure 2:**
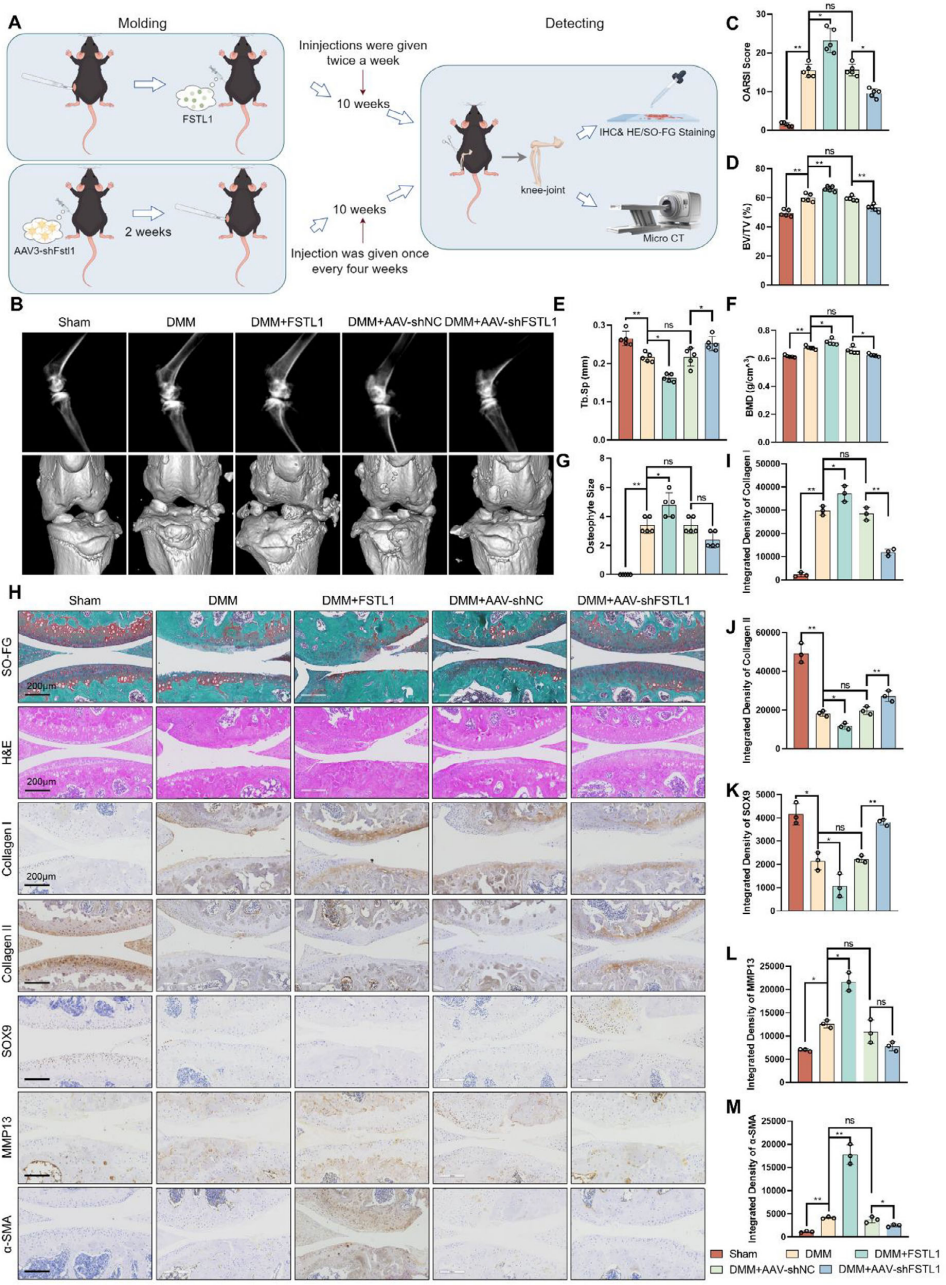
FSTL1 exacerbates the progression of osteoarthritis in DMM mice. A) Schematic diagram of the design and experimental procedure of the mouse model. B–G) Imaging manifestations and the related index analysis of the knee joints in each group of mice. H–M) Results and quantitative analysis of safranin‐O fast green staining, H&E staining, and immunohistochemical staining. ANOVA, *n* = 3, ^*^ *p* < 0.05, ^**^ *p* < 0.01.

To clarify the function of FSTL1 in the development of chondrocyte fibrosis, we generated a mice model of the medial meniscus (DMM). Following the surgical procedure, the mice received intra‐articular injections of either FSTL1 or an interfering lentivirus as a control. Knee tissues were collected 10 weeks postsurgery for further analysis (**Figure** **2**A). To verify the knockdown efficiency of FSTL1 by adeno‐associated virus, we used immunohistochemistry to verify the content of FSTL1 in cartilage and found that the content of FSTL1 in the cartilage layer of the knee joint increased after injection of recombinant FSTL1 protein, while the content of FSTL1 in the cartilage layer was significantly lower in the DMM + AAV‐shFSTL1 group compared to the DMM + AAV‐shNC group (Figure S2A,B, Supporting Information). Based on the images generated from the X‐ray and Micro‐CT, the knee joints in the DMM+AAV‐shFSTL1 group exhibited minimal osteophyte formation, akin to normal joints, whereas prominent osteophyte development was observed in the DMM+FSTL1 group (Figure 2B). In addition, significantly elevated OARSI (the Osteoarthritis Research Society International) scoring of joint cartilage was also observed in mice treated with FSTL1 compared to the control group transduced with the lentivirus targeting FSTL1 (Figure 2C).^[^ ^25^ ^]^ The proliferation of osteophytes and the increase in bone mass in subchondral bone were consistent. CT analysis results showed that FSTL1 significantly increased the trabecular bone volume fraction (BV/TV) and bone mineral density (BMD), and decreased the trabecular bone porosity (Tb.Sp), while these indicators showed the opposite trend in the DMM + AAV‐shFSTL1 group (Figure 2D–G). Furthermore, hematoxylin and eosin (H&E) staining and Safranin O‐Fast Green (SO‐FG) staining indicated that FSTL1 exacerbated cartilage degradation in the joint in contrast to the DMM group. Meanwhile, the downregulation of FSTL1 brought the degree of cartilage wear closer to that of the sham operation group (Figure 2H). After chondrocytes transform from hyaline chondrocytes to fibrocartilage cells, the expression of collagen gradually shifts from type II collagen to type I collagen. With the progression of osteoarthritis, the expression of SOX9 protein—the key transcription factor that maintains chondrocyte homeostasis—declines, while the expression of matrix metalloproteinase 13 (MMP13), α‐smooth muscle actin (α‐SMA), and other proteins that degrade the extracellular matrix and proteoglycans of chondrocytes increases. Immunohistochemical staining of the cartilage verified the reduction in Collagen II and SOX9 levels following FSTL1 treatment, which was reversed by lentiviral injection. Besides, the expression levels of Collagen I, matrix MMP13, and α‐SMA proteins, which serve as markers of inflammation and fibrosis, exhibited an inverse trend of alteration (Figure 2H–M). Taken together, these results suggest that FSTL1 aggravates cartilage erosion and osteophyte production in DMM‐induced mice.

### 2.3 FSTL 1 Induces Fibrosis in Chondrocytes and Glycolysis in Chondrocytes via the HIF‐1 Signaling Pathway

**Figure 3:**
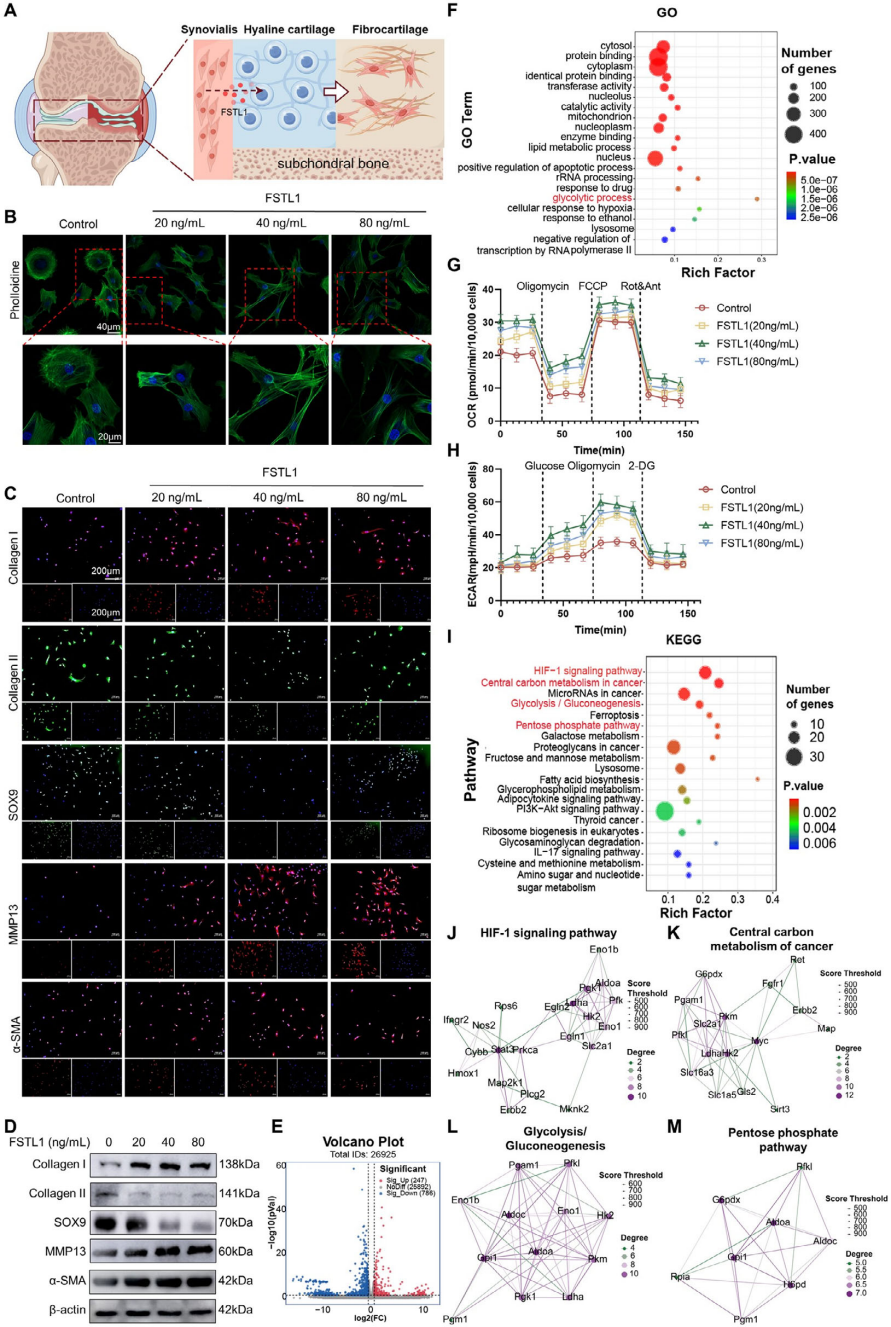
Exogenous FSTL1 promotes chondrocyte and fibrosis glycolysis in chondrocytes via the HIF‐1 signaling pathway. A) Schematic diagram of the mechanism by which FSTL1 induces chondrocyte fibrosis. B) Analysis of chondrocyte morphology (phalloidin staining). C) Analysis of fibrocartilage‐related protein expression (immunofluorescence) stimulated with different concentrations of FSTL1. D) Western blot analysis of fibrocartilage‐related protein levels. E) Volcano plot of differentially expressed genes: blue (downregulated), red (upregulated), gray (no significant difference). F) Analysis of GO enrichment comparing the control group with the group treated with FSTL1. G,H) Results of glycolysis‐related marker detection in chondrocytes. I) KEGG enrichment analysis comparing the control group with the FSTL1‐treated group. J–M) PPI protein interaction network diagram.

Previous investigations by our research group have established that synovial cells exhibit marked upregulation of FSTL1 secretion under arthritic conditions, a mechanism implicated in the pathogenesis of joint degeneration.^[^ ^26^ ^]^ Drawing from prior experimental outcomes, we propose a bold hypothesis: in the progression of arthritis, the transformation of hyaline chondrocytes into fibrocartilage cells is mediated by FSTL1 secreted by synovial cells (**Figure** **3**A).

Based on these findings, we postulate that synovium‐derived FSTL1 may orchestrate the fibrotic transformation of hyaline chondrocytes into fibrocartilage during osteoarthritis progression. To evaluate this premise, chondrocytes were subjected to escalating concentrations of recombinant FSTL1, and the fibrotic transformation was assessed by immunofluorescence microscopy. Phalloidin staining, which selectively binds filamentous actin (F‐actin), revealed dose‐dependent cytoskeletal reorganization, with chondrocytes exhibiting elongated cellular morphologies and increased striated actin structures proportional to FSTL1 concentration (Figure 3B). Concomitant attenuation of hyaline cartilage markers (SOX9, Collagen II) was observed, alongside progressive upregulation of fibrotic markers (Collagen I, α‐smooth muscle actin [α‐SMA]) and the inflammatory protease matrix metalloproteinase‐13 (MMP13), consistent with FSTL1‐mediated fibro‐inflammatory reprogramming (Figure 3C; Figure S3A–E, Supporting Information). Immunoblotting assays corroborated these observations, demonstrating FSTL1‐dependent suppression of chondrogenic markers and induction of fibrotic mediators (Figure 3D; Figure S3F–J, Supporting Information). Intriguingly, maximal fibrotic transformation occurred at 40 ng mL^−1^ FSTL1, with attenuation at higher concentrations, a biphasic response potentially attributable to chondrocyte apoptosis induced by supraphysiological FSTL1 exposure.

To investigate the effects of FSTL1 on chondrocytes, RNA‐Seq was conducted with the treatment of FSTL1 at a concentration of 40 ng mL^−1^ was utilized to stimulate chondrocytes. The results of volcano plot analysis indicated that of the total 26925 genes, 247 genes were significantly upregulated while 786 genes were significantly downregulated (Figure 3E). Upon conducting GO enrichment analysis on all upregulated genes, it was discovered that genes related to glycolysis exhibited the highest enrichment score, suggesting that FSTL1 may significantly promote the expression of glycolysis‐related genes in chondrocytes (Figure 3F).

To further investigate whether FSTL1 enhances the glycolytic capacity of chondrocytes, the extracellular acidification rate (ECAR), oxygen consumption rate (OCR), and lactate production were measured. Consistent with previous results, FSTL1 at a concentration of 40 ng mL^−1^ was found to have the strongest promotive effect on chondrocyte glycolysis (Figure 3G,H; Figure S3K, Supporting Information).

Furthermore, KEGG enrichment analysis of significantly upregulated genes revealed that FSTL1 primarily activates the HIF‐1 signaling pathway in chondrocytes. Simultaneously, genes related to glucose metabolism, encompassing cancer central carbon metabolism, glycolysis/glucose synthesis, and the pentose phosphate pathway, were among the forefront of affected biological processes (Figure 3I). Protein‐protein interaction (PPI) network analysis further indicated that glycolysis‐related proteins such as Ldha, HK2, and Pkm play a pivotal role (Figure 3J–M). These findings suggest that FSTL1 may influence chondrocyte differentiation by mediating the glycolysis process.

### 2.4 FSTL 1 Enhances Glycolysis in Chondrocytes Through the PI3K/AKT/mTOR Signaling Pathway

**Figure 4:**
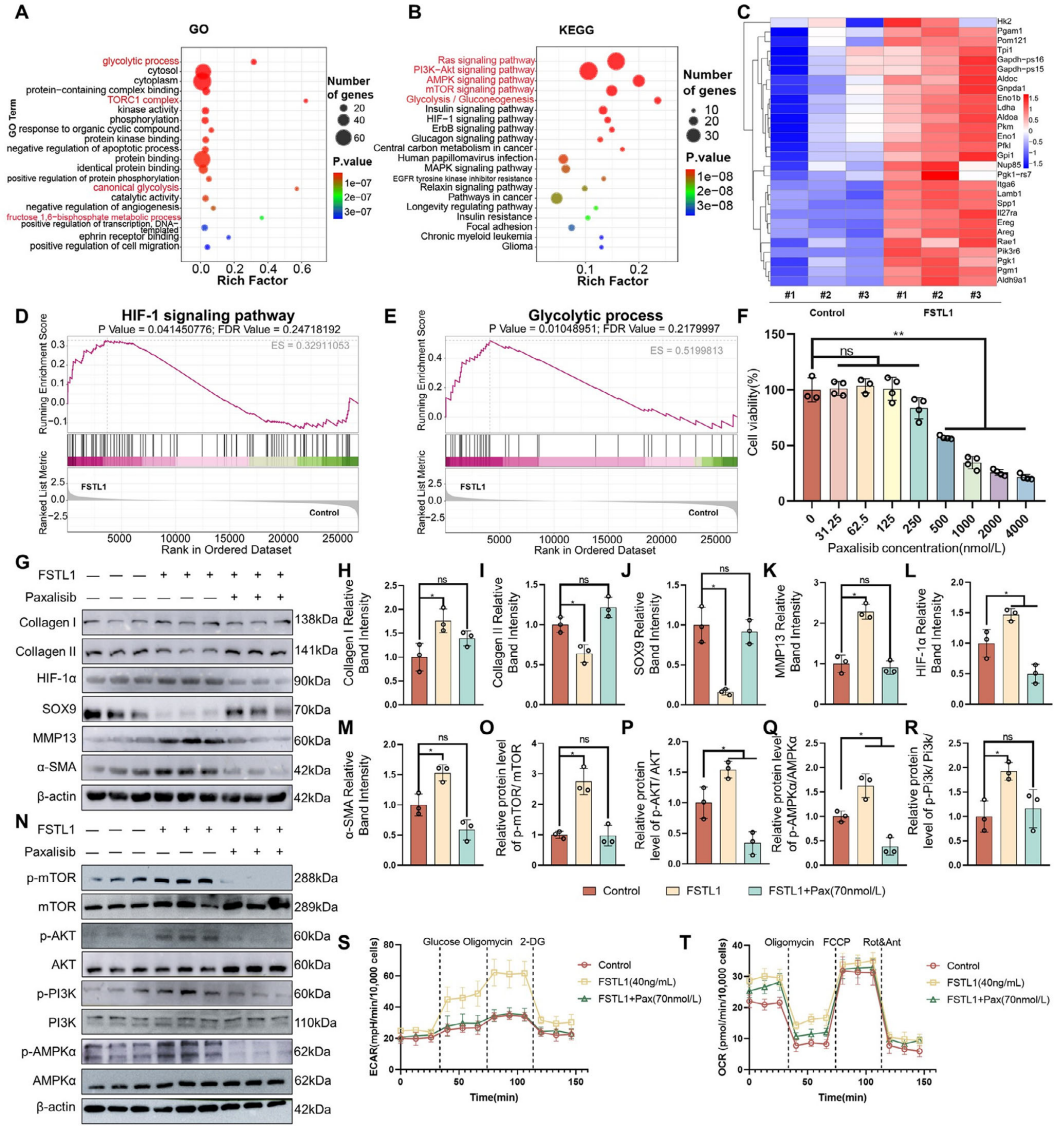
FSTL1 enhances glycolysis in chondrocytes via the PI3K/AKT/mTOR signaling pathway. A,B) GO and KEGG enrichment analysis of genes related to glucose metabolism following FSTL1 treatment. C) Heatmap of glycolysis‐related gene expression. D,E) GSEA enrichment analysis of the HIF‐1 signaling pathway and glycolysis progression after FSTL1 treatment. F) CCK‐8 assay results of paxalisib's cytotoxicity on chondrocytes. G–R) Western blotting results and quantitative analysis of fibrosis‐related proteins and signaling pathways. S,T) Detection results of glycolysis‐related indicators in chondrocytes. ANOVA, *n* = 3, ^*^ *p* < 0.05, ^**^ *p* < 0.01.

To explore how FSTL1 enhances glycolysis in chondrocytes, genes related to sugar metabolism were analyzed. GO enrichment analysis revealed that genes associated with glycolytic process, target of rapamycin complex 1 (TORC1 complex), canonical glycolysis, and fructose 1,6‐bisphosphate metabolic process had high enrichment scores, suggesting that FSTL1 may promote glycolysis and biosynthesis (**Figure** **4**A). KEGG enrichment analysis indicated that FSTL1 primarily activates the Ras signaling pathway. The Ras signaling pathway can activate both the PI3K‐AKT and mTOR signaling pathways (ranked second and fourth, respectively)^[^ ^27^, ^28^ ^]^ and may also influence the AMPK signaling pathway.^[^ ^29^, ^30^ ^]^ Therefore, we verified whether FSTL1 could induce Ras activation and confirmed it does from Western blot results and quantitative analysis (Figure S4, Supporting Information). We hypothesize that FSTL1 primarily activates the PI3K/AKT/mTOR pathway via the Ras signaling rather than the MAPK cascades. This activation subsequently leads to the stimulation of the HIF‐1 signaling pathway (Figure 4B). The heatmap analysis revealed a significant upregulation of glycolysis‐related gene expression in chondrocytes treated with FSTL1 compared to the control group (Figure 4C). GSEA enrichment analysis further confirmed that the HIF‐1 signaling pathway and glycolytic process were significantly activated in the FSTL1 group (Figure 4D,E).

To validate our hypothesis, paxalisib was employed in experimental verification. Paxalisib is an inhibitor that can penetrate the brain and targets PI3K and mTOR, with inhibition constants of 2, 46, 3, 10, and 70 nm for PI3Kα, PI3Kβ, PI3Kδ, PI3Kγ, and mTOR, respectively.^[^ ^31^, ^32^ ^]^ Through the CCK8 cytotoxicity assay, it was discovered that paxalisib concentrations below 125 nmol L^−1^ exerted no significant impact on the proliferative activity of chondrocytes. Consequently, in subsequent experiments, the concentration of paxalisib was set at 70 nmol L^−1^ to ensure the safety and efficacy (Figure 4F).

According to Western blotting results, FSTL1 leads to a reduction in hyaline cartilage characteristic marker proteins SOX9 and Collagen II expression, whereas MMP13, Collagen I, HIF‐1α, and α‐SMA proteins expression rises. Addition of paxalisib significantly inhibited the effects of FSTL1 (Figure 4G–M). Further, the activating effects of FSTL1 on the PI3K‐AKT signaling pathway, mTOR signaling pathway, and AMPK signaling pathway were also inhibited by paxalisib administration (Figure 4N–R). Lastly, the impact of paxalisib on chondrocyte glycolysis was examined. ECAR and OCR detection results showed that paxalisib eliminated the promoting effect of FSTL1 on chondrocyte glycolysis (Figure 4S,T).

These experimental results mentioned above suggest that FSTL1 enhances glycolysis in chondrocytes through the PI3K/AKT/mTOR signaling pathway.

### 2.5 Exogenous Lactate Exhibits a Fibrogenic Effect on Chondrocytes Similar to That of FSTL1

**Figure 5:**
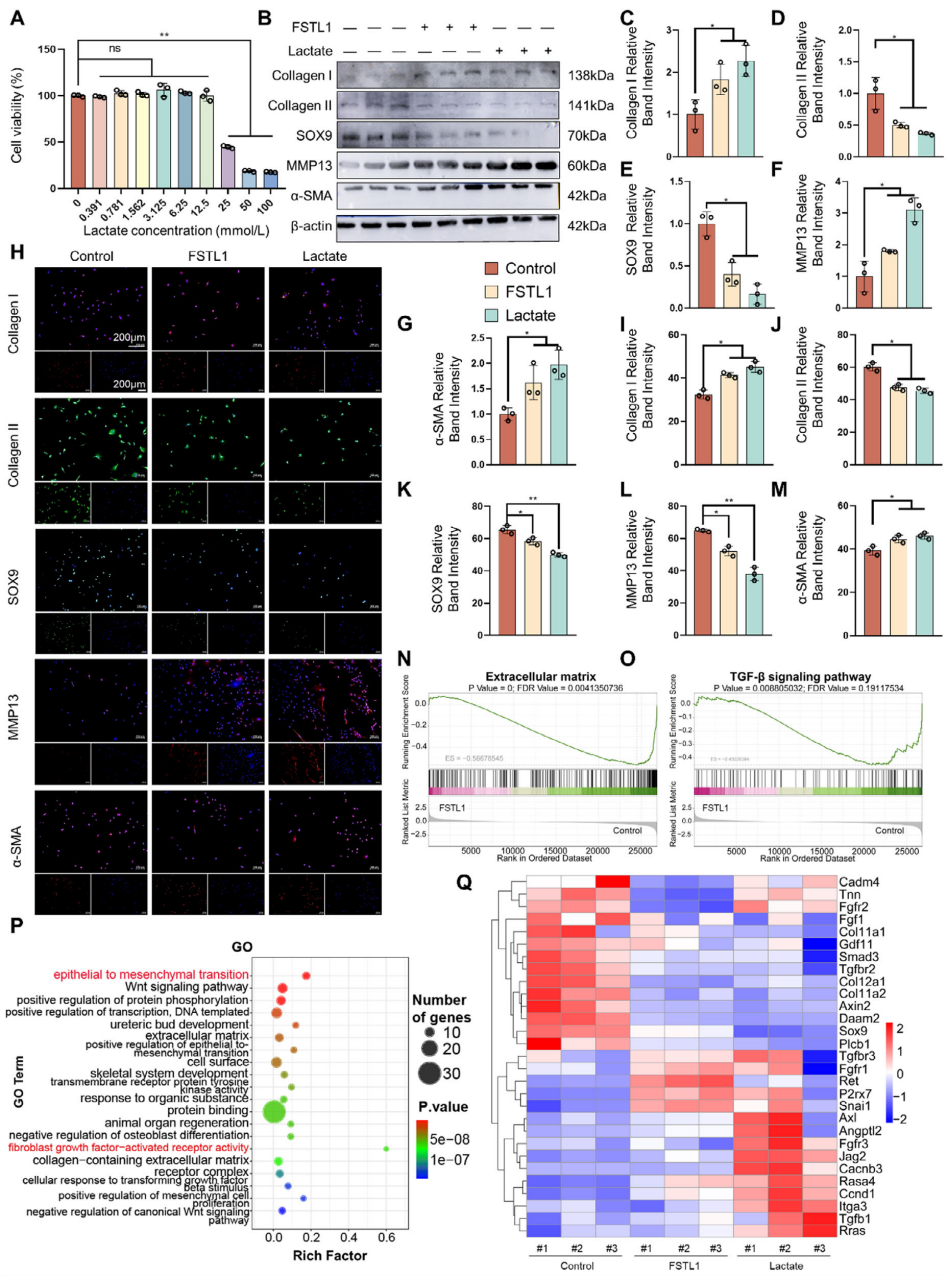
Exogenous lactate mimics the role of FSTL1 to promote chondrocyte fibrosis. A) CCK‐8 assay results for the impacts of lactate on chondrocytes. B–G) Western blotting results and quantitative analysis of fibrosis‐related proteins. H–M) Immunofluorescence results and quantitative analysis of fibrosis‐related proteins. N–O) GSEA enrichment analysis of the control group, FSTL1 treatment group, and lactate treatment group. P) GO enrichment analysis. Q) Heatmap of fibrocartilage‐related gene expression. ANOVA, *n* = 3, ^*^ *p* < 0.05, ^**^ *p* < 0.01.

Due to the significant attenuation of FSTL1's pro‐fibrotic effect on chondrocytes following glycolysis inhibition, the mechanism by which glycolysis promotes chondrocyte fibrosis has been further explored. During glycolysis, the conversion of glucose into lactate is considered the most notable change.^[^ ^33^, ^34^ ^]^ Therefore, exogenous lactate was used to stimulate chondrocytes to verify whether lactate plays a key role within this procedure. Initially, it was determined through the CCK8 assay that lactate concentrations below 12.5 mmol L^−1^ had no significant effect on chondrocyte proliferation. Consequently, the concentration of lactate in subsequent experiments was set at 10 mmol L^−1^ (**Figure** **5**A).

Western blotting results indicated that lactate exhibited similar effects to FSTL1, namely a gradual decrease of the expression of the transparent cartilage characteristic marker proteins SOX9 and Collagen II, while the expression of MMP13, Collagen I, and α‐SMA proteins showed an upward trend (Figure 5B–G; Figure S5A–F, Supporting Information). Immunofluorescence experiments further validated these findings (Figure 5H–M). Subsequently, RNA‐Seq was utilized to compare the gene transcription profiles of the control group, FSTL1 group, and lactate group. By intersecting the upregulated genes in the FSTL1 group and lactate group and conducting correlation analysis, GSEA enrichment analysis suggested that both FSTL1 and lactate downregulated the production of the extracellular matrix of chondrocytes and the TGF‐β signaling pathway (Figure 5N,O). GO enrichment analysis revealed that the enrichment scores for epithelial‐mesenchymal transition of chondrocytes and fibroblast growth factor‐activated receptor activity were high, indicating that both FSTL1 and lactate are involved in the process of chondrocyte fibrosis, but not through the classical TGF‐β pathway, but rather as a specific result caused by lactate (Figure 5P). Heatmaps showed that the expression of fibrocartilage‐related genes was notably increased both in the groups which was treated by FSTL1 and lactate, with a more pronounced effect in the lactate group (Figure 5Q).

These results suggest that the role of FSTL1 in promoting chondrocyte fibrosis may be achieved through the enhancement of glycolysis, leading to lactate accumulation.

### 2.6 FSTL1 Promotes the Upregulation of Genes Related to Chondrocyte Fibrosis Through Histone Lactylation

**Figure 6:**
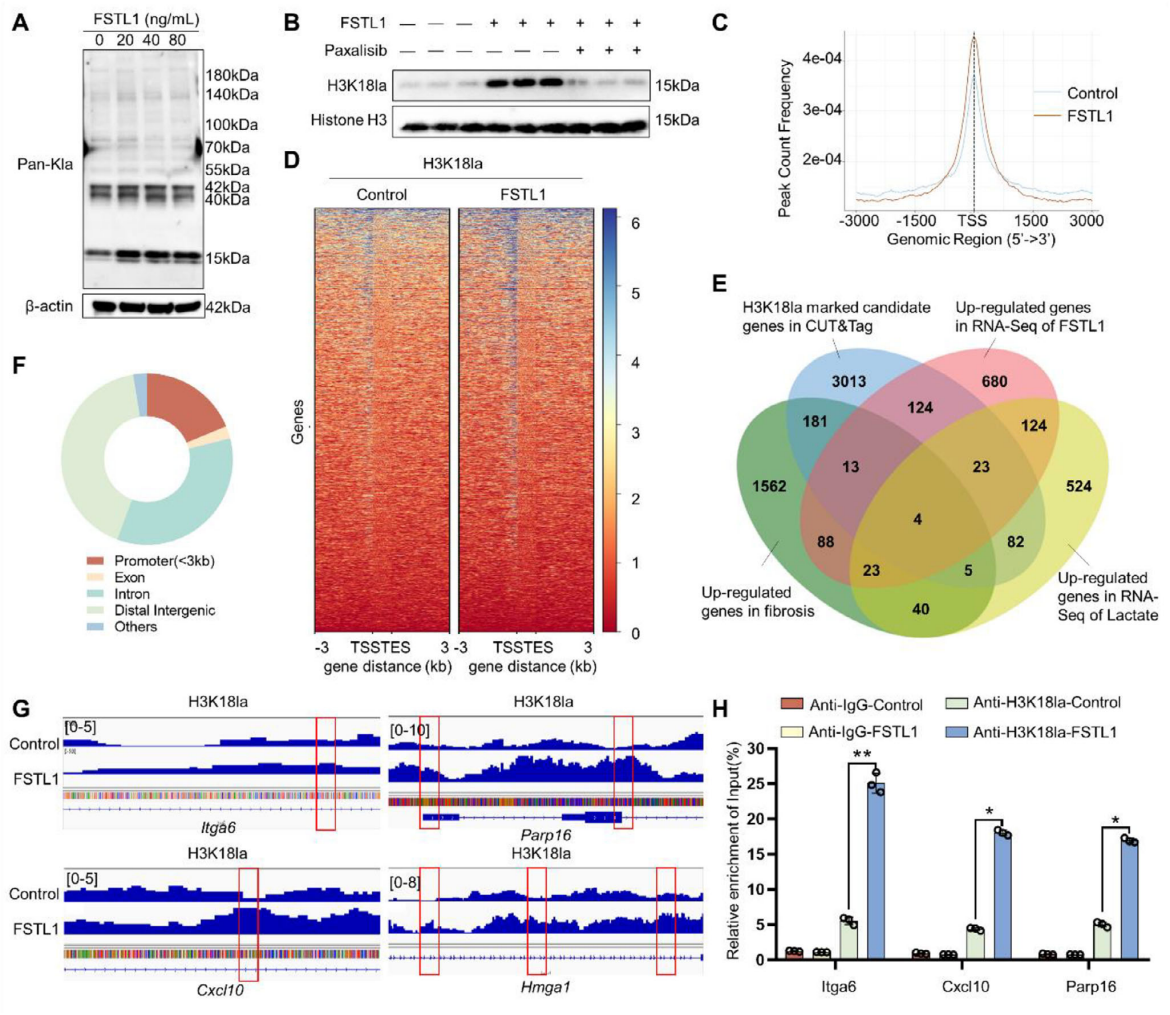
FSTL1 enhances the expression of genes associated with chondrocyte fibrosis via histone lactylation. A) Western blotting results of pan‐lactylation following treatment with different concentrations of FSTL1. B) Western blotting results of H3K18la following treatment with paxalisib. C,D) The heatmaps and curve graphs of genomic occupancy rates of H3K18la in the TSS flanking ±3 kb regions in chondrocytes of the control group and FSTL1 group. E) Venn diagram of upregulated genes in the FSTL1 group and lactate group in RNA‐Seq analysis, as well as H3K18la‐marked genes and fibrosis‐related genes in CUT&Tag. F) Distribution of H3K18la in genomic regions in chondrocytes of the FSTL1 group. G) Normalized peak density of H3K18la on *Itga6, Cxcl10, Parp16, Hmga1* genes, and H) results of ChIP validation experiments. Text, *n* = 3, ^*^ *p* < 0.05, ^**^ *p* < 0.01.

Western blotting was employed to analyze the degree of lactylation at different proteins' lysine residues in chondrocytes under the influence of varying concentrations of FSTL1 (**Figure** **6**A). The results indicated that FSTL1 induces a widespread histone lactylation phenomenon in chondrocytes, with histone 3 lysine 18 lactylation (H3K18la) being the most pronounced, and its extent closely aligns with the expression trend of pan‐histone lactylation and fibrosis‐related proteins (Figure S6A–G, Supporting Information). Within 24 h following the introduction of FSTL1, a significant elevation in the level of H3K18la modification was observed, accompanied by a concurrent increase in fibrin expression levels within chondrocytes. Continuous monitoring revealed that the upward trend in lactylation modification gradually decelerated after 24 h, while fibrin expression levels did not exhibit significant changes. This phenomenon suggests that FSTL1 may influence chondrocyte fibrosis through early epigenetic regulatory mechanisms (Figure S6H–J, Supporting Information). Following the addition of paxalisib treatment, the degree of H3K18la induced by FSTL1 was significantly reduced (Figure 6B; Figure S6K, Supporting Information).

To further elucidate the potential function of H3K18la, a CUT&Tag experiment was conducted. In chondrocytes treated with FSTL1, the H3K18la peaks near the transcription start sites (TSS) were significantly increased compared to the control group (Figure 6C,D). To pinpoint potential genes under the regulation of H3K18la, the intersection of upregulated genes in the FSTL1 group and lactate group from RNA‐Seq analysis, genes with at least a two‐fold increase in H3K18la marker expression in CUT&Tag, and fibrosis‐related genes from Coremine (Sig < 0.05) was taken. The Venn diagram indicated that four genes, namely Itga6, Cxcl10, Parp16, and Hmga1, were common to all four gene sets (Figure 6E). Analysis of the genome‐wide distribution of H3K18la revealed that a significant fraction of histone lactylation modifications were located within the promoter 3 kb region (Figure 6F). Subsequent analysis revealed H3K18la peaks in the promoter regions of the four genes previously mentioned, showing different degrees of increase in the FSTL1 group compared to the control group (Figure 6G). Finally, based on the Ensembl database (mouse, v101 version), enrichment analysis of specific genomic loci revealed that only the promoter regions of *Itga6*, *Cxcl10*, and *Parp16* were identified. The results of ChIP‐qRT‐PCR verified the enrichment of H3K18la at the promoter regions of *Itga6*, *Cxcl10*, and *Parp16*. These results indicated that H3K18la was significantly enriched in the promoter regions of *Itga6, Cxcl10*, and *Parp16* in chondrocytes, which were significantly increased following FSTL1 treatment (Figure 6H). Experimental data show that FSTL1 facilitates chondrocyte fibrosis through histone lactylation, leading to enhanced expression of genes associated with fibrosis.

### 2.7 Pharmacological Inhibition of Lactate Production can Suppress Chondrocyte Fibrosis

Oxamate's inhibitory impact on lactate dehydrogenase (LDH) has been shown to greatly reduce the transformation of pyruvate into lactate.^[^ ^35^ ^]^ Previous study reported that oxamate treatment reduces both the production of lactate and intracellular lactate levels significantly,^[^ ^36^ ^]^ without inducing cell death (Figure S7A, Supporting Information).

**Figure 7:**
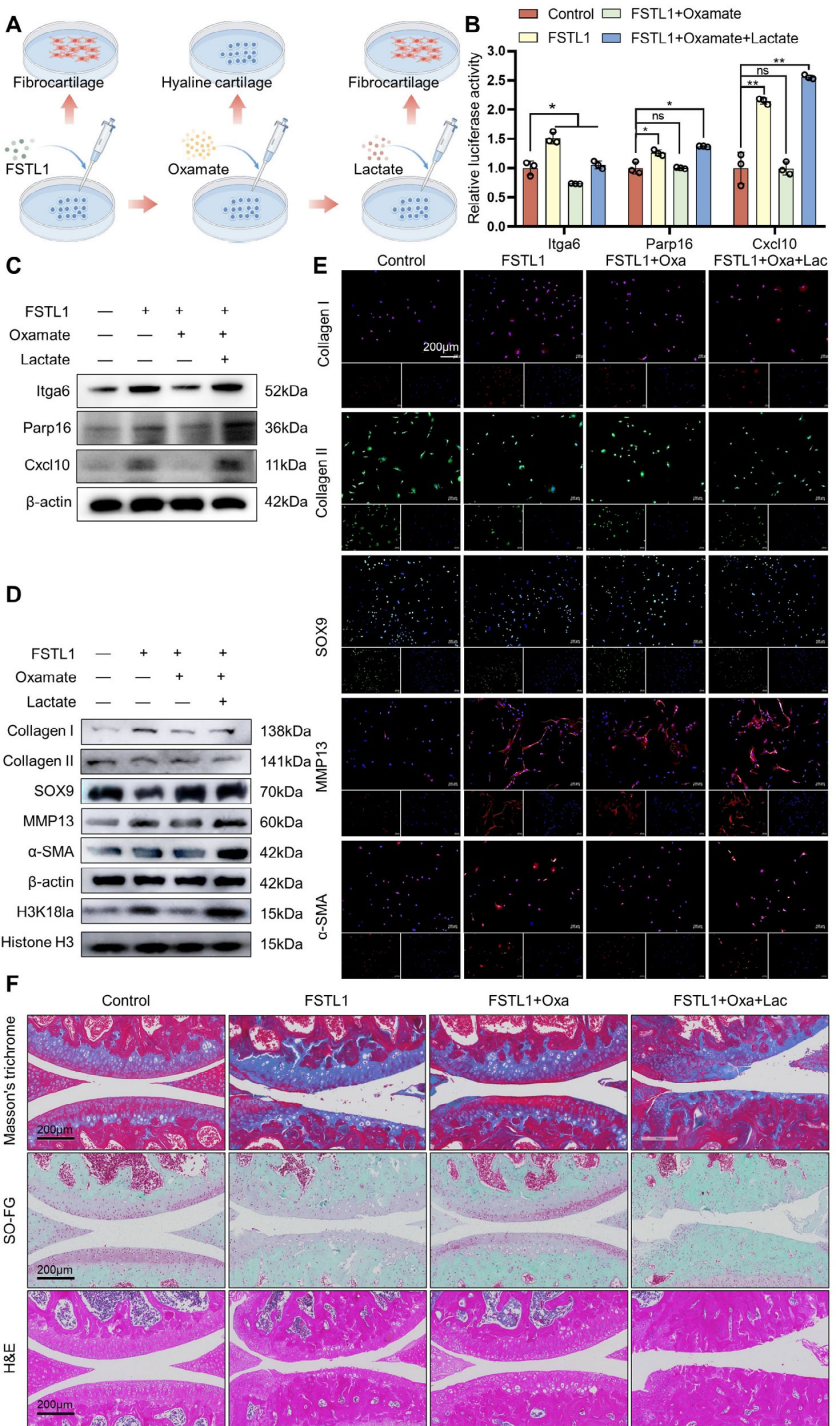
Inhibiting lactate production to alleviate FSTL1‐induced chondrocyte fibrosis. A) Schematic diagram of the design and procedure of the above experiment. B) Dual‐luciferase reporter assay results of *Itga6, Cxcl10, Parp16*. C) Western blotting results of Itga6, Cxcl10, Parp16. D) Western blotting results of fibrosis‐related proteins and H3K18la. E) Immunofluorescence results for proteins related to fibrosis. F) Masson trichrome staining, safranin‐O fast green staining, and H&E staining assay outcomes. Text, n=3, * p<0.05, ** p<0.01.

We found that pharmacological inhibition of lactate production in chondrocytes prevented their fibrotic process, while exogenous lactate could reverse this process (**Figure** **7**A). We first verified the relationship between the presence of lactic acid and the expression of *Itga6, Cxcl10, and Parp16*, and the results suggested that lactate was the key to the upregulation of *Itga6, Cxcl10, and Parp16* expression (Figure 7B,C; Figure S7B–D, Supporting Information).

Furthermore, oxamate treatment has also been found to effectively decrease the expression levels of H3K18la and fibrocartilage‐related proteins. Notably, these reduced expression levels can be restored by exogenous lactate supplementation (Figure 7D; Figure S7E–J, Supporting Information). These experimental results further validated the data from immunofluorescence staining (Figure 7E; Figure S7K–O, Supporting Information).

To further investigate the potential role of lactate in the fibrotic process of osteoarthritis chondrocytes, oxamate was administered to the DMM mouse model to observe its impact on the formation of fibrocartilage on the articular surface. Masson staining, safranin‐O fast green staining, and H&E staining revealed that oxamate significantly alleviated the fibrotic alteration induced by FSTL1 in chondrocytes. However, the therapeutic effect of oxamate was reversed upon intra‐articular injection of lactate (Figure 7F). These findings imply that lactate is a key factor in the fibrotic process of osteoarthritis chondrocytes.

## 3 Discussion

The primary findings of our study exist in that during the progression of OA, FSTL1 enhances glycolysis in chondrocytes by lactate accumulation through activating the PI3K/AKT/mTOR signaling pathway. In turn, the accumulated lactate induces histone lactylation modification and ultimately promoting the expression of genes related to chondrocyte fibrosis to drive chondrocyte fibrosis. This discovery sheds new light on the pathological processes of OA and underscores the significant role of FSTL1 in chondrocyte fibrosis.

First, through comparative analysis of scRNA‐seq data of chondrocytes from normal individuals and OA patients, we observed a significant increase in fibrocartilage cell populations in OA. Research on cartilage wear and regeneration are available,^[^ ^37^ ^]^ yet studies on phenotypic changes in chondrocytes, particularly the differentiation mechanisms of fibrocartilage cells, are absent. The functional and mechanical properties of fibrocartilage cells differ significantly from those of hyaline cartilage, potentially serving as one of the key drivers of OA disease progression.^[^ ^38^ ^]^ Second, FSTL1 is commonly regarded as an inflammatory mediator in the progression of arthritis,^[^ ^18^ ^]^ as the positive correlation of FSTL1 with the progression of OA has been clinically validated.^[^ ^24^ ^]^ This correlation is also confirmed experimentally in this study (Figure 1). In previous experiments, FSTL1 was observed to induce chondrocyte fibrosis, leading to the speculation that it may be one of the key drivers of chondrocyte fibrosis in OA. This hypothesis was confirmed through additional cell and animal experiments, highlighting the important role of FSTL1 in chondrocyte fibrosis. The mechanism of action of FSTL1 in OA differs from its reported functions in other organs.^[^ ^39^, ^40^, ^41^ ^]^ In OA, FSTL1 is primarily secreted by synoviocytes, rather than being expressed in increased amounts by chondrocytes themselves. We found that the FSTL1 concentration in the majority of patients was below 40 ng mL^−1^. Out of curiosity, we wanted to explore the consequences of higher concentrations of FSTL1. However, the results did not show that the effect of FSTL1 gradually increased with concentration, and the FSTL1 concentration range in previous clinical data did not include 80 ng mL^−1^, so we did not conduct in‐depth research on why the effect of 80 ng mL^−1^ FSTL1 was slightly lower than that of 40 ng mL^−1^. The RNA‐Seq data analysis results indicate that among the glycolysis‐related genes upregulated by FSTL1, the genes associated with the TORC1 complex were the most highly correlated (Figure 4). The targets of the TORC1 signaling pathway are widely recognized as key regulators of growth and metabolism,^[^ ^42^ ^]^ further supporting the phenomenon observed in previous experiments that FSTL1 promotes chondrocyte proliferation. Through KEGG enrichment analysis, the genes upregulated by FSTL1 primarily affect the HIF‐1 signaling pathway (Figure 4). In the KEGG enrichment analysis of glycolysis‐related genes, the top‐ranked signaling pathways, such as the Ras/PI3K‐AKT/mTOR signaling pathway, were found to regulate the HIF‐1 signaling pathway.^[^ ^43^, ^44^, ^45^ ^]^ Additionally, the Ras signaling pathway has been confirmed to be the upstream regulator of the PI3K‐AKT and mTOR signaling pathways.^[^ ^46^ ^]^ Based on these findings, we therefore speculated that FSTL1 may regulate the fibrosis process of chondrocytes through the Ras/PI3K/AKT/mTOR/HIF‐1 axis. Subsequently, our speculation was confirmed by experiments using paxalisib.

In fibrotic tissues, the abnormal accumulation of lactic acid has not only been confirmed as a result of metabolic reprogramming but also been found to directly regulate gene expression through epigenetic mechanisms.^[^ ^47^ ^]^ Recent studies have shown that lactic acid can serve as a substrate to induce histone lactylation modification, thereby reshaping the transcriptional activity of pro‐fibrotic genes at the chromatin level.^[^ ^6^ ^]^ For instance, in a pulmonary fibrosis model, lactic acid produced by myofibroblast glycolysis is transported to macrophages via paracrine action, subsequently inducing the occurrence of histone H3K18 lactylation. This modification has been proven to activate the promoter regions of pro‐fibrotic genes such as TGF‐β and VEGFA.^[^ ^48^ ^]^ This mechanism reveals the direct association between metabolic products and epigenetic modifications in the progression of fibrosis. RNA‐Seq data analysis further indicated that FSTL1 primarily drives fibrosis through lactate accumulation rather than other biological processes, a conclusion verified by experiments involving lactate production inhibition and exogenous lactate supplementation (Figure 6). This study demonstrates that FSTL1 induces chondrocyte fibrosis by promoting glycolysis and lactate accumulation in chondrocytes, rather than through traditional inflammatory pathways.^[^ ^49^ ^]^ Although lactic acid may cause changes in cellular physiological processes through non‐specific effects (such as pH changes), the regulation of chromatin is mainly through lactylation modification.^[^ ^50^ ^]^ The subsequent experimental results showed that the trend of histone lactylation changes was consistent with the changes in chondrocyte fibrosis also indirectly proved this conclusion. As the histone lactylation modification has not been documented to be directly involved in chondrocyte fibrosis before. Our study unveils the pivotal role of histone lactylation in chondrocyte fibrosis, which might offer a new direction for the further investigation of fibrosis mechanisms.

However, this study also exhibits certain limitations: firstly, due to the relatively limited availability of synovial fluid from non‐OA patients, most samples were obtained from individuals with traumatic knee injuries. Matching these samples with those from OA patients in terms of age, gender, and body weight proved challenging; consequently, only preliminary screening was conducted, and the resulting conclusions should be interpreted with caution. Second, in exploring the mechanism by which FSTL1 promotes glycolysis, only the pathways with the most significant differences were validated, without conducting in‐depth experimental verification of other potential genes screened out by the PPI network. Finally, in the study of histone lactylation modification, we only conducted a preliminary screening of histone lactylation sites, without comprehensively screening more histone and nonhistone sites. Future research should further explore these potential targets and their roles in fibrosis.

In summary, our study unveils a novel mechanism by which FSTL1 regulates chondrocyte fibrosis through lactate accumulation and histone lactylation. The enhancement of glycolysis by FSTL1 and lactate‐mediated histone modification plays a decisive role in chondrocyte fibrosis. These findings suggest that interventions targeting FSTL1 or lactate metabolism may emerge as potential strategies for treating chondrocyte fibrosis, which provide new insights into the treatment of OA.

## 4 Experimental Section

### Strains, Reagents, and Instruments

The sources for FSTL1 and sodium oxamate were MedChemExpress, while ZUNYAN provided the Cell Counting Kit‐8 (CCK‐8). Avantor Life Sciences (Shanghai, Co., Ltd.) supplied the fetal bovine serum (FBS). The DMEM‐F12 (Dulbecco's Modified Eagle Medium/Nutrient Mixture F‐12) was obtained from HyClone Laboratories in Logan, USA, whereas lactate and Triton X‐100 were acquired from Sigma‐Aldrich in St. Louis, USA. Moreover, the Extracellular Acidification Rate Assay Kit and the Oxygen Consumption Rate Assay Kit were acquired from Bestbio. The ELISA kit was purchased from MLBio; the EDTA decalcification solution and trypsin digestion solution were obtained from Solarbio; and the serum‐free protein cryopreservation solution was purchased from New Cell & Molecular Biotech. The primary antibodies were purchased from ABclonal, Proteintech, and other suppliers. Antibody Dilution Buffer was purchased from Elabscience Biotechnology. For details, please refer to the antibody table in the supplementary methods.

### Study Populations

Synovial tissue case data were extracted from the electronic medical record system of the Third Affiliated Hospital of Nanjing Medical University from January 2015 to December 2024. The inclusion criteria were adult patients diagnosed with traumatic knee injuries or knee osteoarthritis, with complete preoperative laboratory tests and imaging reports. Exclusion criteria included patients in pregnancy, those with a history of other systemic malignancies, and cases with missing key clinical data. As research on glycolysis‐related aspects of chondrocytes was later conducted, patients with endocrine diseases such as diabetes and hyperthyroidism, or metabolic diseases, were excluded. Ultimately, 60 eligible subjects were consecutively included in the analysis. Since age is an important factor influencing OA, when comparing tissues of different OA severities, we used tissues from patients aged between 55 and 65 to minimize the impact of disease duration on individual differences as much as possible. Multidimensional clinical parameters, including demographic characteristics (age, height, weight) and treatment response data (K‐L score and Vas score), were systematically collected. Data were independently entered by two individuals and cross‐verified through the hospital information management system. Inconsistencies were resolved through arbitration by a clinical expert panel. Retrospective data were anonymized, and sensitive information (such as ID numbers, contact details) was desensitized and stored. The research protocol was approved by the Clinical Research Ethics Committee of Changzhou Second People's Hospital (approval number: [2024]KY218‐01). Informed consent was waived due to the use of surgical waste tissue and the absence of potential risks. All data were encrypted and stored on the hospital's internal server, with access restricted to authenticated research team members. Data export and analysis complied with the relevant regulations of the “Ethical Review Measures for Human Life Sciences and Medical Research Involving Human Subjects.”

### Cell Culture

The ATDC5 cells, obtained from the European Collection of Authenticated Cell Cultures, were cultured in DMEM‐F12 with the addition of 10% fetal bovine serum, 1% penicillin‐streptomycin, and 1% insulin‐transferrin‐selenium to induce chondrogenic differentiation.^[^ ^51^ ^]^ Differentiated chondrocytes should avoid excessive passage, and should not be used after two passages, as chondrocytes tend to dedifferentiate and lose their characteristics during the passage process. For FSTL1 treatment, the cells were treated on day 1. On day 3, the cells received treatments with 50 mmol L^−1^ oxamate (HY‐W013032A, MCE), 70 mmol L^−1^ paxalisib (GDC‐0084, MCE), or 10 mmol L^−1^ lactate (252476‐100G, Sigma), and were subsequently collected on day 5.^[^ ^39^ ^]^ All drugs were pH‐neutralized prior to administration.

### Animal Experiments

Three‐month‐old male C57BL/6 wild‐type mice, weighing 25–30 grams, were acquired from Shanghai Slac Laboratory Animal Co., Ltd. All animal experiments and care procedures complied with the guidelines and regulations set by the Institutional Animal Care and Use Committee of Zhejiang Center of Laboratory Animals (ZJCLA‐IACUC‐20011099). The mice were kept in an environment set at 25 °C with a 12‐h cycle of light and darkness. Before the experiments, the animals were given a week to adjust to a specific diet. OA was caused surgically by destabilizing the medial meniscus (DMM) in the right knee joints.^[^ ^52^ ^]^ These surgeries were performed on the right knee joints of 3‐month‐old male wild‐type (WT) littermate mice. Thirty mice were randomly assigned to five groups: the sham group, the DMM group, the DMM + FSTL1 group, the DMM+ adeno‐associated virus (AAV)shNC group, and the DMM+AAVshFSTL1 group. All drug injections were administered intra‐articularly. Drug administration commenced following the surgeries, with all drugs prepared in a biosafety cabinet to ensure sterile post‐articular cavity injection. The recombinant mouse FSTL1 protein was injected twice a week. The adeno‐associated virus was injected once every four weeks, with injections at 2 weeks before surgery, 2 weeks after surgery, and 6 weeks after surgery. The knee joints were harvested 10 weeks postsurgery. Afterward, the samples were preserved in 10% zinc‐formalin for 48 h and then decalcified using EDTA solution (Solarbio, China) for a month before being embedded in paraffin. The experiment followed the Declaration of Helsinki guidelines and received approval from the Institutional Animal Ethics Committee at Nanjing Medical University.

### Measurement of the ECAR and OCR

ATDC 5 cells were seeded in black‐wall 96‐well plates at a concentration of 8 × 10^^4^ cells per well and left to incubate for 24 h. After incubation, the culture medium was discarded, and the cells were rinsed twice with assay buffer. Fluorescent probes were then introduced, and the cells were stimulated with varying concentrations of FSTL1. To measure the oxygen consumption rate (OCR), an additional oxygen‐sealing liquid was required. Following this, the extracellular acidification rate (ECAR) and OCR were tracked and documented every 3–5 min for a duration of 2 h using a fluorescent microplate reader.

### ChIP‐qRT‐PCR

ATDC5 cells were treated with 40 ng mL^−1^ FSTL1 for three days, following which the cells were harvested for ChIP‐qRT‐PCR experiments. The ultrasonic treatment conditions were set at 10 s of sonication followed by a 20‐s pause, with a total cycle duration of 1 min and 40 s. The remaining experimental procedures were conducted in accordance with the instructions provided in the Beyotime ChIP Kit (P2080S).

## Conflict of Interest

The authors declare no conflict of interest.

## Author Contributions

Y.W. and F.L. were responsible for the study's conception and design. F.L. and Y.Y. were involved in data acquisition and interpretation. F.L. and Y.T., with help from G.Y., H.H., S.L., Y.L., M.L., and L.W., handled data cleaning, statistical analysis, and model development. F.L., C.X., G.Z., and B.Z. collected and processed the clinical samples. F.L. and H.H. wrote the initial draft of the manuscript. Y.W. played a role in the critical revision of significant intellectual content. Every author participated in the significant revision of the manuscript for essential intellectual content and gave their final approval for the version to be published.

## Supporting information



Supporting Information

## Data Availability

The data that support the findings of this study are available from the corresponding author upon reasonable request.
