# Supplementary material for: FSTL1 Orchestrates Metabolic‐Epigenetic Crosstalk: Glycolysis‐Dependent H3K18 Lactylation Drives Cartilage Fibrosis in Osteoarthritis
Source: Adv Sci (Weinh). 2025 Nov 26;13(8):e12002. doi: 10.1002/advs.202512002 (PMC12884748; doi:10.1002/advs.202512002)
Supplement: Supplementary file 1 — Supporting Information [file ADVS-13-e12002-s001.docx]

**Supplementary Figures**

**Supplementary Figure 1**

**
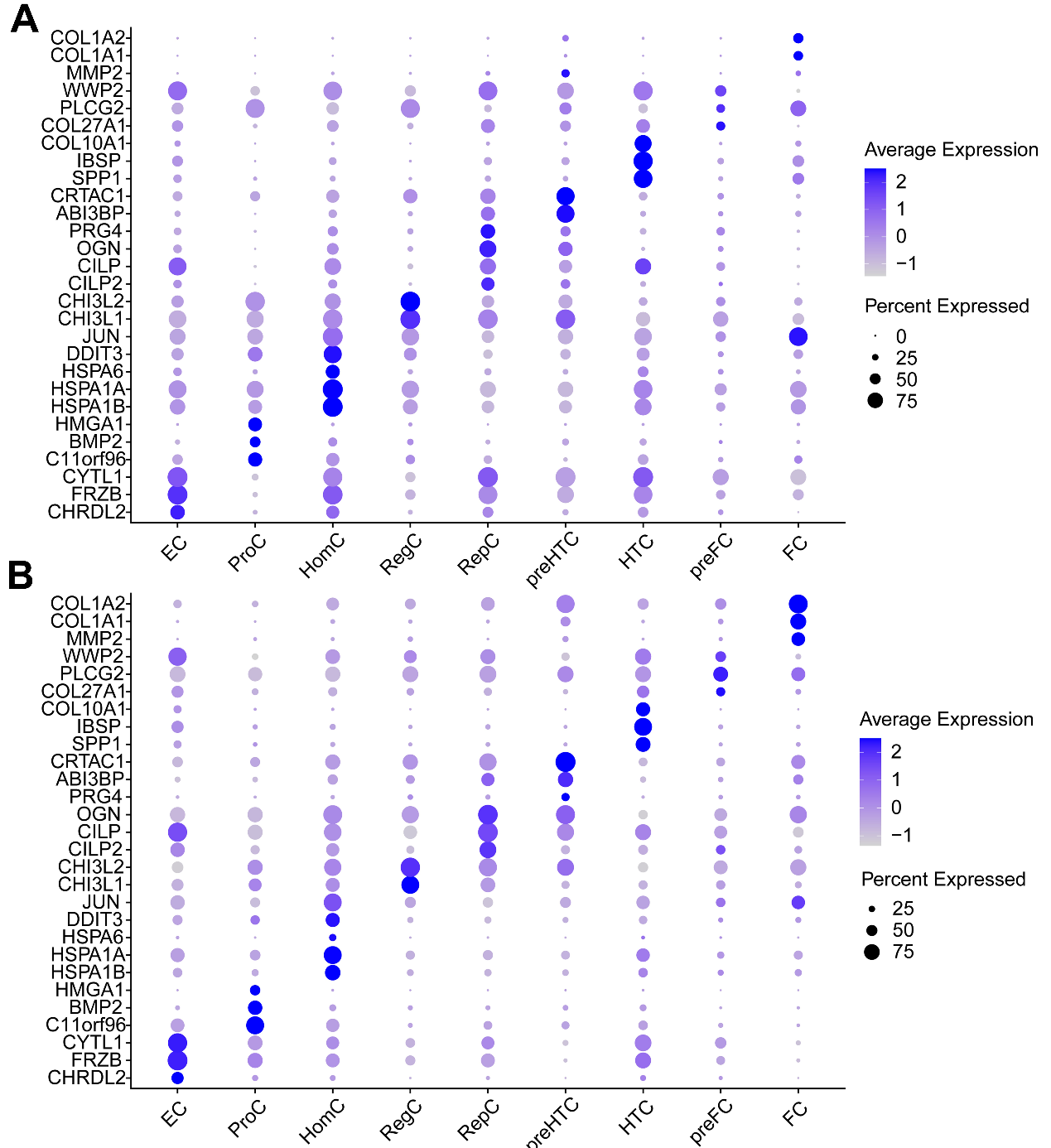
**

**Figure S1.** Cluster analysis of single-cell sequencing data from chondrocytes of healthy (A) and OA patients (B).

**Supplementary Figure 2**

**
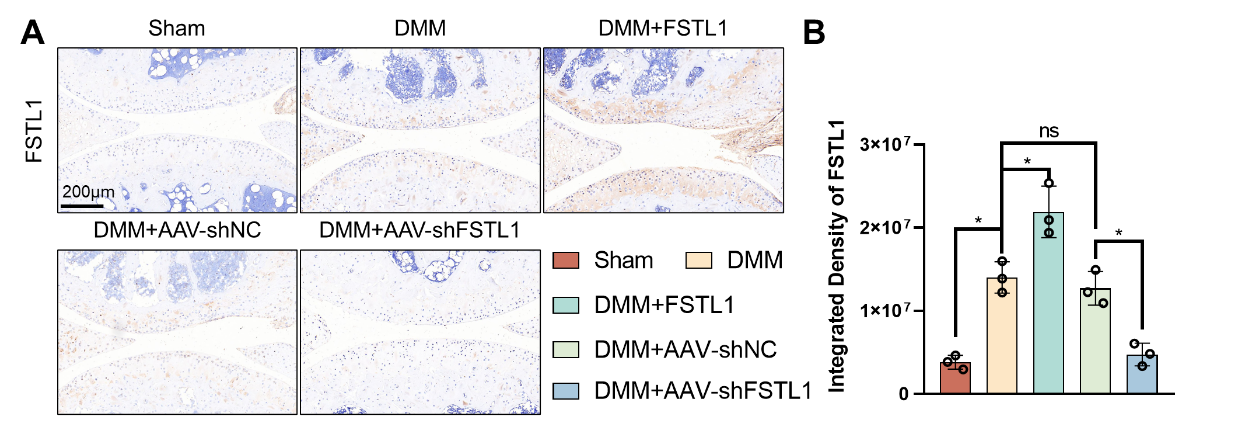
**

**Figure S2.** FSTL1 exacerbates the progression of osteoarthritis in DMM mice. ANOVA, n=3, * p<0.05, ** p<0.01.

**Supplementary Figure 3**


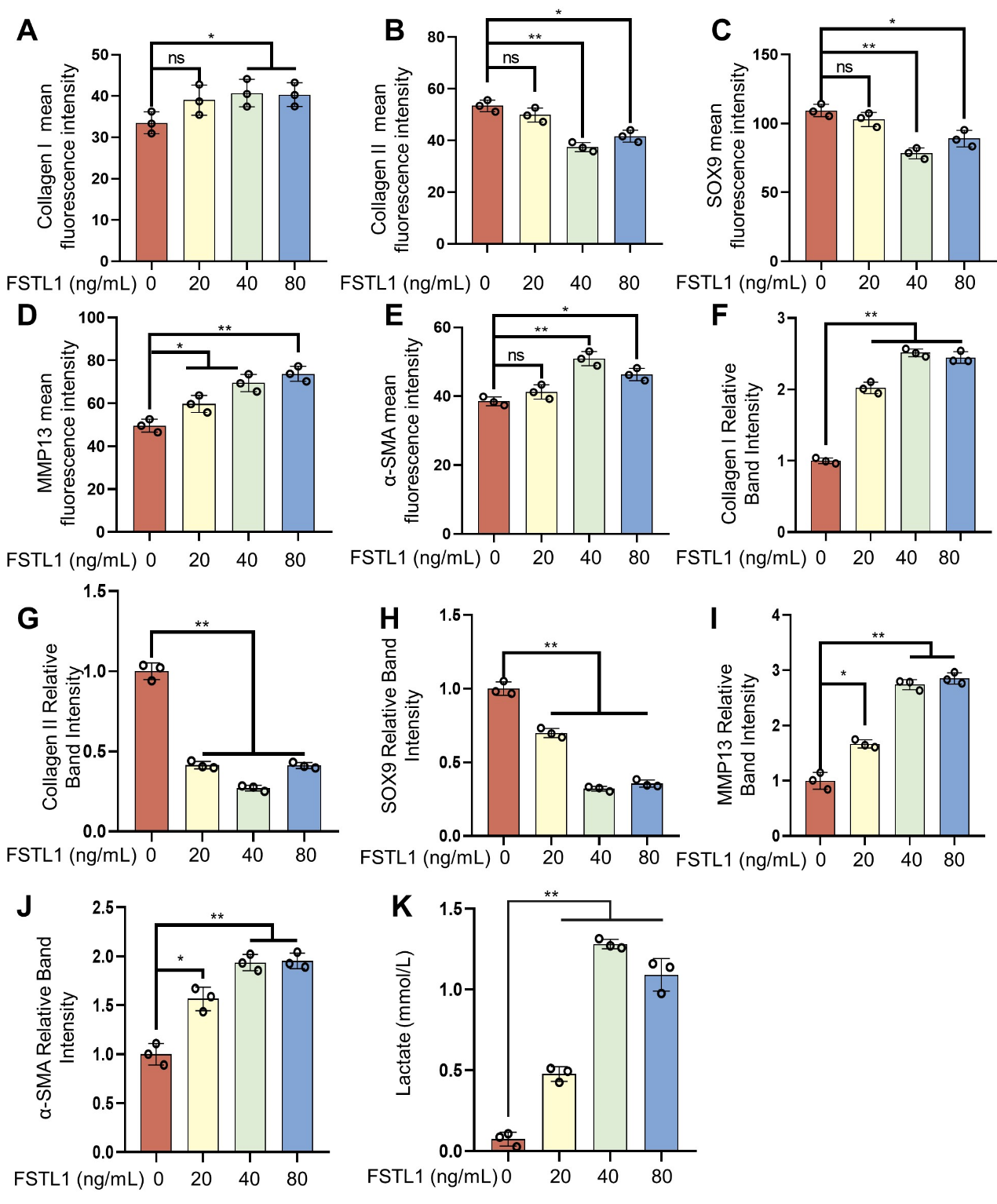


**Figure S3.** Quantitative analysis of Immunofluorescence (A-E) and Western blot (F-J) for fibrocartilage-related protein expression under the stimulation of different concentrations of FSTL1. (K) Quantitative analysis of lactate generation in chondrocytes under the stimulation of different concentrations of FSTL1. ANOVA, n=3, * p<0.05, ** p<0.01.

**Supplementary Figure 4**

**
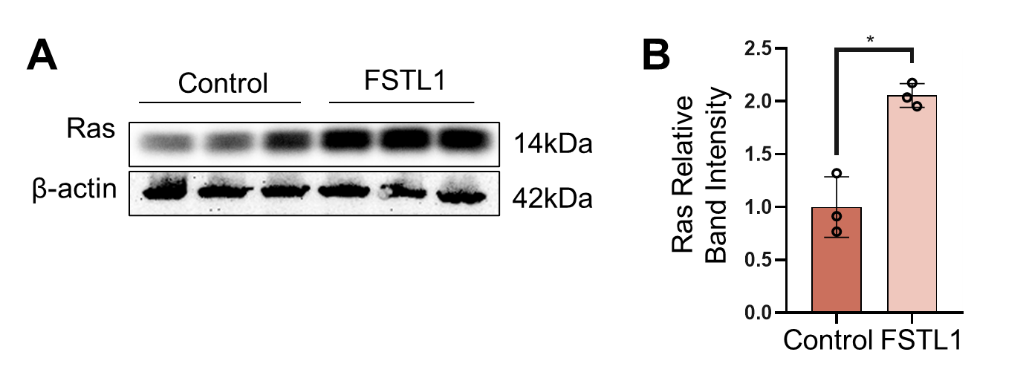
**

**Figure S4.** Western blotting results and quantitative analysis of Ras. Text, n=3, * p<0.05, ** p<0.01.

**Supplementary Figure 5**


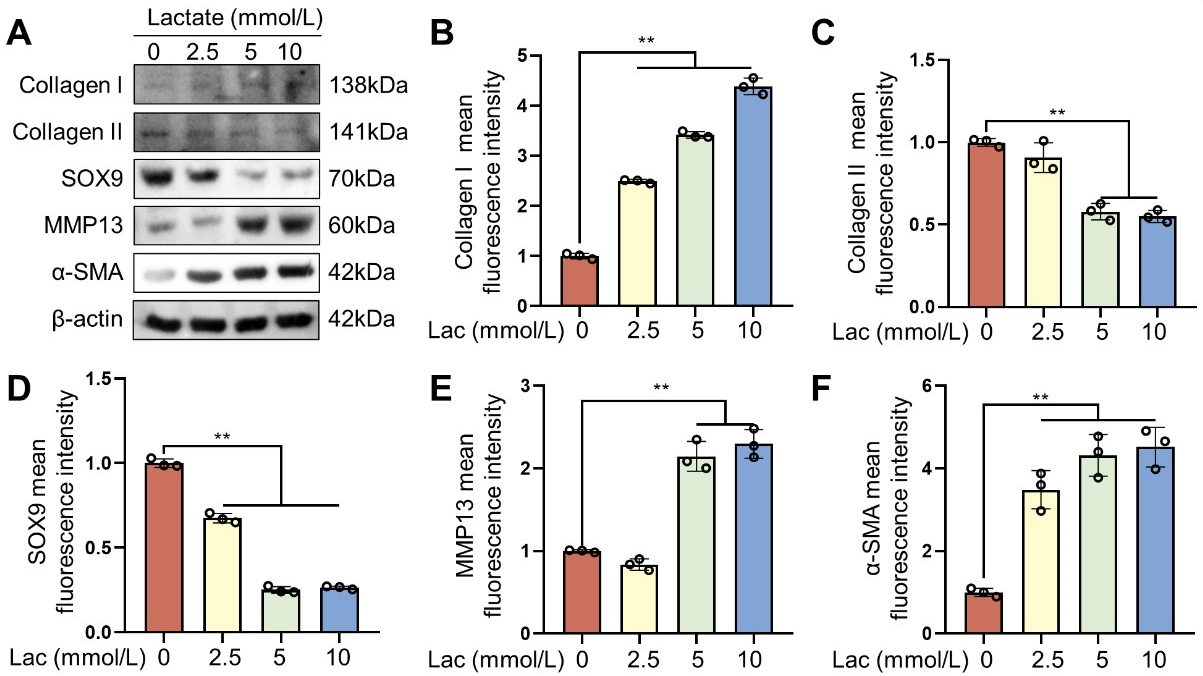


**Figure S5.** (A-F) Quantitative analysis of fibrocartilage cell-related protein expression under stimulation of varying concentrations of FSTL1. ANOVA, n=3, * p<0.05, ** p<0.01.

**Supplementary Figure 6**

**
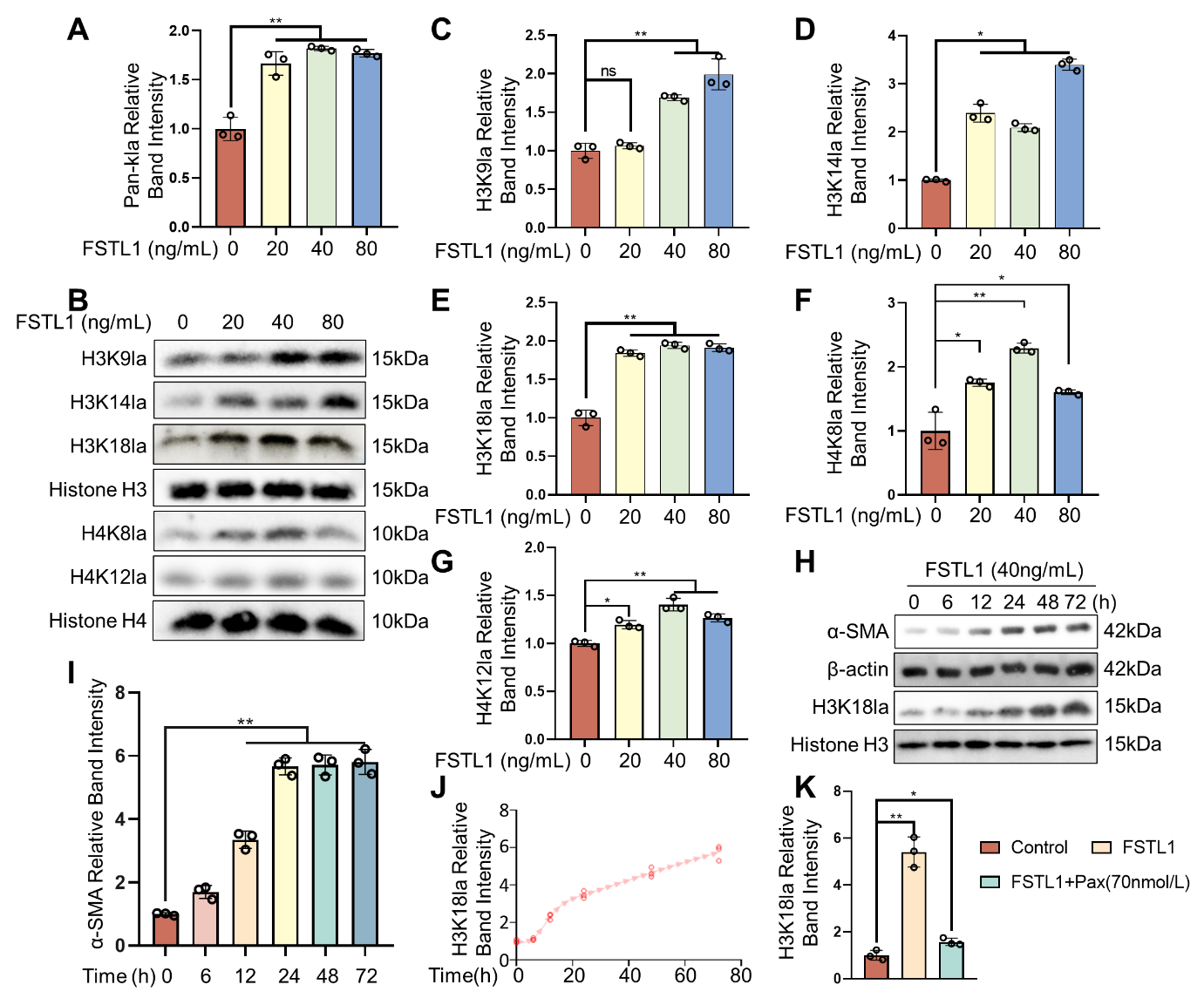
**

**Figure S6.** **(A-G)** Quantitative analysis of the degree of lactylation in different histone groups and pan-histone lactylation of chondrocytes under stimulation by FSTL1 at varying concentrations. (H-J) Western blotting analysis of the time-dependent increase in α-SMA and H3K18la expression following FSTL1 treatment of chondrocytes. (K) Western blotting analysis of H3K18la following treatment with paxalisib. ANOVA, n=3, * p<0.05, ** p<0.01.

**Supplementary Figure 7**

**
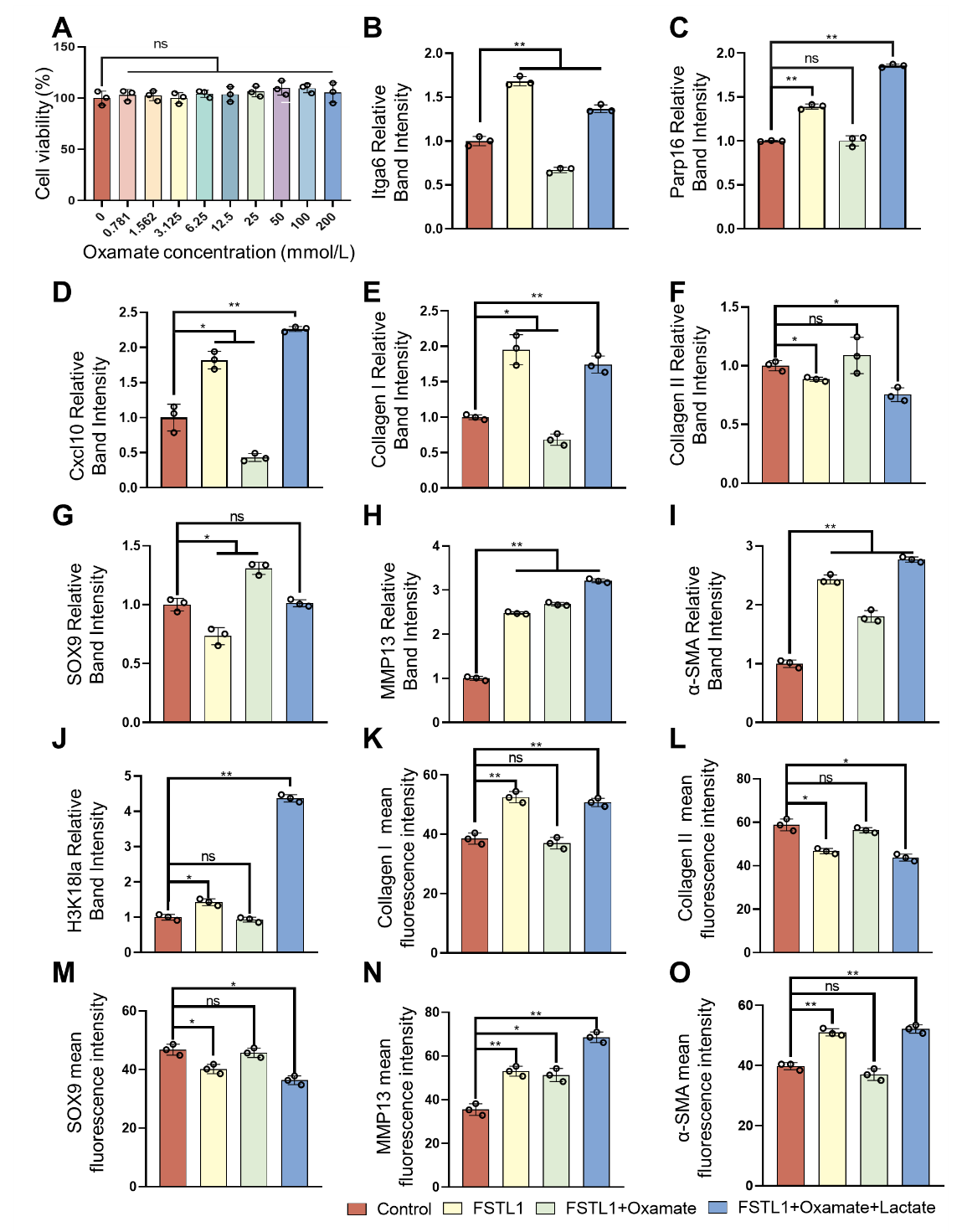
**

**Figure S7.** (A) CCK-8 assay results for the cytotoxicity of lactate on chondrocytes. (B-D) Western blotting and quantitative analysis results of Itga6, Cxcl10, Parp16. (E-J) Western blotting and quantitative analysis results of fibrosis-related proteins and H3K18la. (K-O) Immunofluorescence and quantitative analysis results for proteins related to fibrosis. ANOVA, n=3, * p<0.05, ** p<0.01.

**Supplementary Methods**

**Table1: Antibody Table**

| Reagent or Resource | Source | Identifier |
| --- | --- | --- |
| Antibodies |  |  |
| Aggrecan | Cell Signaling Technology | #P16112 |
| COL1A1 | Cell Signaling Technology | #P02452 |
| P38 MAPK Antibody | Cell Signaling Technology | Q16539,P53778, Q15759 |
| Phospho-SAPK/JNK (Thr183/Tyr185)(G9)Mouse mAb | Cell Signaling Technology | P45983 |
| Phospho-p38MAPK (Thr180/Tyr182)(D3F9)XP Rabbit mAb | Cell Signaling Technology | #Q16539 #Q15264 #P53778 #Q15759 |
| SAPK/JNK Antibody | Cell Signaling Technology | P45983 |
| Phospho-PI3Kinase p85(Tyr458)/p55(Tyr199)(E3U1H) Rabbit mAb | Cell Signaling Technology | P27986 Q92569 O00459 |
| P44/42 MAPK (Erk1/2)(L34F12) Mouse mAb | Cell Signaling Technology | #P27361,#P28482 |
| Phospho-Akt (Ser473)(193H12) Rabbit mAb | Cell Signaling Technology | P31751 Q9Y243 P31749 |
| AKT Antibody | Cell Signaling Technology | P31751 Q9Y243 P31749 |
| GAPDH (D4C6R)Mouse mAb | Cell Signaling Technology | #P04406 |
| Anti-ADAMTS5 antibody | Abcam | AB41037 |
| COL2 | Cloud-Clone Corp | RPD194Mu01 |
| Phospho-MEK1(MAP2K1) Rabbit Polyclonal Antibody | Origene | TA325636 |
| MTOR Rabbit Polyclonal Antibody | Origene | AP06628PU-N |
| Phospho-MTOR Rabbit Polyclonal Antibody | Origene | TA325698 |
| MEK1(MAP2K1) Rabbit Monoclonal Antibody | Origene | R08-6A7 |
| HIF-1 alpha(HIF1A) Rabbit Polycional Anitibody | Origene | AP20633PU-N |
| Anti-L-Lactyl-Histone H3(Lys18) Rabbit mAb-ChIP Grade | PTM Biolabs | Cat#PTM-1427RM |
| Histone H3 Mouse McAb | proteintech | 68345-1-Ig |
| Beta Actin Monoclonal antibody | proteintech | 66009-1-Ig |
| Smooth muscle actin specific Monoclonal antibody | proteintech | 67735-1-Ig |
| CXCL10/IP-10 | ABclonal | A27370 |

**Table2: Promoter sequence of** **genes related to chondrocyte fibrosis**

| **Genes** | | **The position of the promoter sequence on the genome** |
| --- | --- | --- |
| **Mouse-Cxcl10--promoter-684bp** | TTTTGAACCGGTACACTCCAGGCTCCTGTCCCATTCCC  AGGATGAGTCACCTCCATCCCCTTCATCACTGTCACCT  CTATGCGAGATCTATGTAACCTCACCTACTCTTCCAGG  TCCCAGGTATCTGCTGCCTTGGGTGCTGTGCAGAGTGA  CATCCACACTCACTTTCATCCTTGACCCTGTAACCACA  CACTCACAAAGAAGACAATCAAAGCATCCCTGAGAGA  ATCAGCACGGGGCAACAATTAGCTTTGCAATTCCTCTT  GTAACTTGTAAACA**TTAGTGTGACACTTCCGGCTTC**  TGTTCTGAATGCCTGCTCTAACTGTTCACTAAGTTGTA  TA**G**ACTTTGGAT**CTGGCTAAATTTGGCGTGTGAT**TT  TTTTTTAAAGTTTATTCCAAGTATTTTATAATAAAGCA  TATGAAGTAAAAAGCAAAAATAAACAGTGTAAACAC  AACAAATGTAAAAACCATGGTTAGAACCTGACTTAGA  TATCAGTTCTGTGTTTTATTTATGAGAGAAGGAAGATG  AGAATTAAAGCCATTTCCAGACTTATTCTGCAAGGCA  CTGCATCTGATTTCTCAAACAGCTCACGCTTTGGAAAG  TGAAACTTACCTCACTCGTTAAAAATTAAAAGGAGCA  CAAGAGGGGAGAGGGAAATTCCAAGTTCATGGGTCA  CAATAAACACAA | |
| **Mouse-Itga6-promoter-415bp** | CACCTGTAACTGAAGTACTTGGTGGTGGAGTCAAGAG  GATAATGAGTTCAAGGTCATCTTCAACTATCTATGGA  GTTCAAGGCCAGATTTCCATAGGAAGCAATGACAATC  TTCCAAGAGATTCTGTTAGGAGTCTGAGAGTTTGACTC  AACAC**CAGGGATGTTTGGCTTCCTAGG**CAGGAAAC  AGGTGGAAGACAGACAGGCACAA**C**AACTGATAGTTTC  TTACCCAGTACGGGCATATTCCAGAACAAGCGTC**TCA**  **GCCTTCTTCAGACCAGATT**TCATCTCTACAAGTACC  ATTCGATTTGCACTGTGCATTTGTAATATTTAAATTTA  ATTTACTGTAATTTAAATGTCAACTTGAGGATTGAAA  TCAAGAAGGTAACAATGACCATTCATGTATTATTTGC  CAATATGTC | |
| **Mouse-Parp16--promoter-244bp** | CCTTCCAATGGGAATTAACCACAGGGAAACAGTCT  TTGGGACTCTGACACAATGGGTGAGCTCAGGACCC  A**TTCCATTCCGATCTGAGATGTGTG**TGTTTCCGG  TCCTTTCTGGAACATTC**T**GTGTTTGCCTCCCTCAAG  GCCTGACACCCACACCTCTGCCCTAACAAGGC**CTT**  **CATGACTCTTGGGCAGACT**GCTCCCACCACCCTCC  ACAAAGCGCTTGCCTGCTAGCATGCATGAGACTG | |

**Subpopulation Analysis of Chondrocytes**

A comprehensive analysis was conducted on chondrocytes from three healthy samples and sixteen OA samples sourced from the GEO database's GSE255460 project. Utilizing the FindSubcluster function within the Seurat package, further analysis of chondrocyte subpopulations was performed. Subsequently, dimensionality reduction analysis (RunPCA, k=30) and clustering analysis (FindCluster, based on the Leuven algorithm, resolution=1.2) were applied to identify distinct chondrocyte subpopulations, which were validated through corresponding marker genes. The visualization of scRNA-seq data was achieved via the UMAP algorithm.

**Cell viability assay**

The survival rate of cells subjected to specified concentrations of oxamate and paxalisib was assessed utilizing the CCK-8 cell proliferation/cytotoxicity assay kit. ATDC 5 cells were planted in 96-well plates at a concentration of 1×10^3^ cells per well. After the cells adhered, the culture medium was switched to one with the specific drugs. Following a 48-hour incubation, the medium was discarded, and 100 μL of blank medium with 10% CCK-8 solution was added to each well, as per the kit's guidelines. The plates were then incubated for 2 hours at 37°C. In the end, a microplate reader from Thermo Fisher Scientific in Waltham, MA, was used to measure the absorbance of each well.

**Histological and histomorphometric analysis**

Mouse joints were fixed, decalcified, paraffin-embedded, sectioned, and dewaxed according to previously established protocols. The sections were treated with hematoxylin-eosin, Safranin O-Fast Green, or underwent immunohistochemistry. To retrieve antigens, the samples were incubated in a preheated trypsin solution at 37°C for 15 minutes, then rinsed with tap water for 10 minutes. The sections were exposed to 0.3% hydrogen peroxide for 10 minutes, followed by two 10-minute rinses in TBS with 0.025% Triton X-100. Following this, the sections were blocked with TBS containing 1% BSA for 2 hours at room temperature. Next, a primary antibody diluted in 1% BSA (1:50) was added dropwise to the slides until the tissue was fully covered. The sections were kept in an incubation box at 4°C overnight and subsequently rinsed twice for 10 minutes each in TBS containing 0.025% Triton X-100. A secondary antibody, also diluted in 1% BSA (1:50), was applied until the tissue was completely covered. The sections were placed in an incubation cassette and kept at 37°C for an hour. Following this, they were rinsed three times for 5 minutes each in TBS before applying the Streptavidin-Biotin Complex (SABC) for a 35-minute incubation. DAB color development solution was used under light protection, and the reaction was monitored microscopically to halt color development at the appropriate time. After dehydration and mounting, images were captured using a light microscope. Ultimately, different authors calculated the OARSI score for the knee, and ImageJ software was employed to analyze images and measure the brown-stained areas.

**Immunofluorescence**

ATDC5 cells were placed in 6-well plates and exposed to suitable concentrations of FSTL1. Afterward, the cells were fixed using 4% paraformaldehyde (PFA) for 15 minutes at room temperature and then permeabilized with 0.2% Triton X-100 for 30 minutes. Following this, the cells were thoroughly washed with PBS and blocked with 1% bovine serum albumin (BSA). Incubation with the relevant primary antibody (1:50) was conducted overnight at 4°C, followed by staining with fluorescently labeled secondary antibodies for 1 hour. In cases where cytoskeletal morphology was to be observed, the cells were incubated with ghost pen cyclopeptide staining solution for 30 minutes. In the end, all cells were stained with DAPI for 10 minutes, then observed and photographed using immunofluorescence microscopy.

**Western blot analysis**

RIPA lysate supplemented with PMSF was employed to lyse ATDC5 cells and extract total protein. For the extraction of histones, further cell disruption was achieved through sonication using an ultrasonic cell crusher for a duration of 5 minutes. The protein samples that were extracted were then centrifuged at 4°C and 12,000 rpm for 15 minutes. The protein concentration was measured and adjusted using the BCA Protein Assay Kit. The protein samples were separated using polyacrylamide gel electrophoresis with gels made from the PAGE Gel Fast Preparation Kit and then transferred to polyvinylidene fluoride (PVDF) membranes. The PVDF membranes were subjected to a blocking procedure using skimmed milk for a duration of two hours at ambient temperature. Afterward, they were left with the primary antibody at 4°C overnight. The next day, the membranes were treated with secondary antibodies for an hour at room temperature. Protein expression was then detected utilizing an ultrasensitive enhanced chemiluminescence (ECL) kit, and the protein bands were quantified employing ImageJ software.

**RNA sequencing and analysis**

RNA was initially extracted from the cells utilizing the TRIZOL reagent (ATDC 5 _ Ctrl versus ATDC 5 _ FSTL 1 and ATDC 5 _ Lactate; n = 3 for each group). Following RNA quality assessment, the samples were dispatched to Hangzhou Lianchuan Biotechnology Co., Ltd. for RNA sequencing and subsequent differential expression analysis via their corporate cloud platform. Genes with a P-value below 0.05 were considered differentially expressed and underwent further analysis using Gene Set Enrichment Analysis (GSEA), along with Gene Ontology (GO) and Kyoto Encyclopedia of Genes and Genomes (KEGG) enrichment analyses. The other two groups of cells (ATDC5_Ctrl and ATDC5_FSTL1) were frozen and sent to Hangzhou Lianchuan Biotechnology Co., Ltd. for CUT & Tag analysis. All data and results of CUT & Tag were obtained from the services provided by the company.

**Safranin O-fast green staining**

The sections were successively treated in xylene I (8 min), xylene II (8 min), absolute ethanol I (6 min), absolute ethanol II (6 min), 95% ethanol (6 min), 85% ethanol (6 min), and 75% ethanol (5 min), and then rinsed with running water. Subsequently, freshly prepared Weigert's hematoxylin solution was dropped onto the sections and stained for 3-5 min, followed by water washing. Acid differentiation solution was used for differentiation for 15 s, and then rinsed with distilled water for 10 min. Fast green staining solution was used for staining for 3-5 min by dropping or immersion. After staining, a weak acid solution was quickly used to wash the sections for 10-15 s to remove residual fast green. After air drying, safranin O staining solution was used for staining for 1-2 min, and absolute ethanol was used to wash off the excess staining solution. Finally, the sections were dehydrated with absolute ethanol, cleared with xylene, and mounted with neutral resin.

**Masson's trichrome staining**

The sections were dewaxed as described in the previous Safranin O-fast green staining method. Then, Weigert's iron hematoxylin staining solution was used for staining for 5-10 min, followed by thorough water washing. Masson's staining solution was used for staining for 3-5 min, and then rinsed with distilled water for 1 min. Subsequently, aniline blue staining solution was used for staining for 5-10 min. A weak acid working solution was prepared with a volume ratio of distilled water to weak acid solution of 2:1 and used to wash the sections for 1 min. Without water washing, the sections were directly transferred to the aniline blue staining solution for staining for 1-2 min. Finally, 95% ethanol was used for rapid dehydration, absolute ethanol was used for three dehydrations (5-10 s each), xylene was used for three clearing treatments (1-2 min each), and neutral resin was used for mounting.

**X-ray and micro-CT**

The mouse knee joint tissues were fixed in 10% neutral buffered formalin for 24 hours. Subsequently, an X-ray detector (parameters: 20-100 kV, 20 W, <5 μm microfocus, 6-position metal filter) and a Bruker microCT SkyScan 1276 high-resolution in vivo micro-CT system were used for scanning. The scanning parameters were set as follows: voltage 55 kV, current 200 μA, filter AI 0.25 mm, pixel size 6 μm, rotation step 0.3°. Image reconstruction and analysis were performed using NRecon software.

**Elisa**

The synovial fluid was digested with 0.05% hyaluronidase (Beyotime, ST1386-25mg) at 37℃ for 5 minutes before detection to reduce viscosity and enhance protein release efficiency, thereby improving detection accuracy. The subsequent ELISA operation was strictly carried out according to the kit instructions (mlbio, RX102996H-96T).

**Plasmid Construction and Cell Transfection**

The sequences of the fragments enriched by the H3K18la CUT-tag antibody on the Itga6, Cxcl10, and Parp16 promoters were cloned into the pMCS-Fluc-SV40-hRluc-Neo vector (MIAOLING PLASMID, P38699) to construct the dual-luciferase reporter plasmids. The overexpression plasmids of Itga6, Cxcl10, and Parp16 were purchased from Genscript. The genomic positions of the relevant enriched sequences are listed in Supplementary Table 2. Cell transfection was performed using Lipofectamine 2000 (Invitrogen, Carlsbad, CA, USA) according to the manufacturer's instructions.

**Dual-Luciferase Reporter Gene Assay**

After transfection, the cells in 12-well plates were lysed with 300 μL of 1× cell lysis buffer (Promega) per well. 15 μL of the cell lysate supernatant was transferred to a 96-well microplate, and the activities of firefly and Renilla luciferases were detected according to the reagent instructions (MeilunBio, MA0518-1) using a fluorescence microplate reader. Renilla luciferase was used as an internal reference, and the luciferase activity ratio (Firefly Luciferase/ Renilla Luciferase) was used for result calculation.

**Statistical analysis**

Statistical analyses were conducted utilizing GraphPad Prism software (version 8.0). Data are expressed as the mean ± standard deviation (SD). Paired or independent sample t-tests were used to compare the average values between two groups, while one-way ANOVA and the rank-sum test were applied for additional comparisons. To evaluate the statistical significance of mean values across several variables, a multivariate analysis of variance (MANOVA) was used. A p-value below 0.05 was deemed statistically significant.
